# Teneurin4 dimer structures reveal a calcium‐stabilized compact conformation supporting homomeric trans‐interactions

**DOI:** 10.15252/embj.2020107505

**Published:** 2022-01-31

**Authors:** Dimphna H Meijer, Cátia P Frias, J Wouter Beugelink, Yanthi N Deurloo, Bert J C Janssen

**Affiliations:** ^1^ Department of Bionanoscience Kavli Institute of Nanoscience Delft University of Technology Delft The Netherlands; ^2^ Department of Chemistry Faculty of Science Structural Biochemistry Bijvoet Center for Biomolecular Research Utrecht University Utrecht The Netherlands

**Keywords:** neuroscience, structural biology, synaptic cell adhesion, Teneurins, transmembrane proteins, Neuroscience, Structural Biology

## Abstract

Establishment of correct synaptic connections is a crucial step during neural circuitry formation. The Teneurin family of neuronal transmembrane proteins promotes cell–cell adhesion via homophilic and heterophilic interactions, and is required for synaptic partner matching in the visual and hippocampal systems in vertebrates. It remains unclear how individual Teneurins form macromolecular *cis*‐ and *trans*‐synaptic protein complexes. Here, we present a 2.7 Å cryo‐EM structure of the dimeric ectodomain of human Teneurin4. The structure reveals a compact conformation of the dimer, stabilized by interactions mediated by the C‐rich, YD‐shell, and ABD domains. A 1.5 Å crystal structure of the C‐rich domain shows three conserved calcium binding sites, and thermal unfolding assays and SAXS‐based rigid‐body modeling demonstrate that the compactness and stability of Teneurin4 dimers are calcium‐dependent. Teneurin4 dimers form a more extended conformation in conditions that lack calcium. Cellular assays reveal that the compact *cis*‐dimer is compatible with homomeric *trans*‐interactions. Together, these findings support a role for teneurins as a scaffold for macromolecular complex assembly and the establishment of *cis*‐ and *trans*‐synaptic interactions to construct functional neuronal circuits.

## Graphical abstract

The Cryo‐EM structure of the ectodomain of Teneurin4 shows a compact dimer conformation that supports trans‐synaptic interactions.

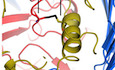

## Introduction

During central nervous system (CNS) development, neural circuitry formation involves complex interactions of molecular guidance and recognition cues. Members of the Teneurin protein family function as such cues and promote cell‐cell adhesion through homophilic and heterophilic interactions (Rubin *et al*, 2002; Silva *et al*, 2011; Hong *et al*, 2012; Mosca *et al*, 2012; Beckmann *et al*, 2013; Boucard *et al*, 2014; Berns *et al*, 2018; Sando *et al*, 2019; Pederick *et al*, 2021). Teneurin proteins are large evolutionarily conserved dimeric type II transmembrane proteins, with an intracellular domain that is responsible for downstream signaling (Rubin *et al*, 2002; Tucker *et al*, 2012). Early works in *Drosophila* have shown that Ten‐a and Ten‐m function as homophilic matching cues in the olfactory bulb and in the neuromuscular junction (Hong *et al*, 2012; Mosca *et al*, 2012). In mammals, Teneurin has four paralogs that are predominantly expressed in the brain, with partially overlapping expression patterns from early embryonic development to adulthood (Ben‐Zur *et al*, 2000; Zhou *et al*, 2003). Teneurins have been shown to play crucial roles in the development of retinal, hippocampal, and cortical circuits. In fact, recent mouse genetic experiments have revealed that the concomitant axonal and dendritic expression of Teneurin3 promotes the correct neuronal pathfinding and wiring in the mouse hippocampus (Berns *et al*, 2018; Pederick *et al*, 2021).

Besides homophilic interactions, Teneurin proteins can also establish heterophilic interactions with a member of the adhesion G‐protein‐coupled receptors, known as Latrophilin (Silva *et al*, 2011; Boucard *et al*, 2014; Vysokov *et al*, 2016; Li *et al*, 2020; Pederick *et al*, 2021). Latrophilins themselves have been shown to interact with fibronectin leucine‐rich repeat transmembrane proteins (FLRTs; O'Sullivan *et al*, 2012; Lu *et al*, 2015; Ranaivoson *et al*, 2015; Jackson *et al*, 2016). Coincident interaction of Latrophilin with its two binding partners (Teneurin and FLRT) potentiates the formation of excitatory synapses in the mouse hippocampus (Silva *et al*, 2011; Boucard *et al*, 2012; Sando *et al*, 2019). Notably, interfering with the Latrophilin–Teneurin interaction specifically has resulted in a decrease in excitatory synapse formation (Li *et al*, 2020). More recently, Del Toro *et al*. have established an additional role for Teneurin2 in complex with Latrophilin and FLRT in cortical cell migration *in vitro* and *in vivo* (Del Toro *et al*, 2020).

In humans, Teneurins have been associated with specific neuronal disorders, such as essential tremor, microphthalmia, general anosmia, schizophrenia, and bipolar disorder (Burbach & Meijer, 2019), highlighting the biomedical need to better understand how Teneurins function and orchestrate the formation of neuronal networks.

In the recent years, structural characterizations of the partial ectodomain of Teneurin2 and Teneurin3 by crystallography and cryo‐electron microscopy have revealed an extracellular ~1,900 residue superfold, with no resemblance to other typical cell‐adhesion proteins (Jackson *et al*, 2018; Li *et al*, 2018). The C‐terminal region of the ectodomain is folded into a large barrel‐shaped structure, termed YD‐shell, with a beta‐propeller NHL domain positioned at an almost 90° angle. The barrel is sealed upstream by a so‐called fibronectin plug domain and capped downstream by its own inward spiraling C‐terminal. The YD shell, together with the NHL domain, resembles a bacterial toxin system known as TcB and TcC of *Y. entomophaga* and *P. luminescens,* now known to be present in other bacterial strains as well (Jackson *et al*, 2018, 2019). Two additional domains, the antibiotic‐binding like (ABD) and Tox‐GHH domains, are located at the ultimate C‐terminal region and appended onto the YD shell. Finally, the transthyretin (TTR)‐like domain is the most N‐terminal domain of the superfold and wedges in between the FN‐plug and NHL domain. Eight predicted epidermal growth factor (EGF)‐like domains are located upstream of the Teneurin superfold. EGF2 and EGF5 each harbor a free cysteine that enable covalent *cis‐*dimerization. Interaction of a confined region on the YD‐shell, opposite of the ABD and Tox‐GHH domains, with Latrophilin, possibly in *trans* (opposite cells), might form the basis of a ternary complex consisting of Teneurin, Latrophilin, and FLRT connecting the pre‐ and postsynaptic membrane (Del Toro *et al*, 2020; Li *et al*, 2020). Interestingly, Teneurin‐Latrophilin binding is prohibited by an NHL splice insert version of membrane‐bound Teneurin (Boucard *et al*, 2014; Li *et al*, 2020).

So far, detailed structural characterizations dealt with monomeric versions of Teneurin proteins (Jackson *et al*, 2018; Li *et al*, 2018, 2020; Del Toro *et al*, 2020). However in biological systems, Teneurins are expressed on the cell surface as covalently bound dimers by means of two disulfide bonds in the extracellular EGF‐like repeats 2 and 5 (Feng *et al*, 2002). How would macromolecular complex assembly further be supported by the homodimeric conformation of Teneurins? Using cryo‐electron microscopy, X‐ray diffraction (XRD), small angle X‐ray scattering (SAXS), and thermostability assays, supported by cell biological assays, we show here that dimeric Teneurin4 can adopt a compact architecture for complex assembly allowing both *cis‐* and *trans*‐interactions. We identify three calcium‐binding sites in the previously unreported C‐rich domain and show that calcium stabilizes the compact Teneurin4 ectodomain conformation. The dimeric nature of Teneurin might be instrumental for the clustering of cell adhesion complexes to establish functional circuits in the developing brain.

## Results

### Covalent dimers of human Teneurin4 are compact molecules

**Figure 1:**
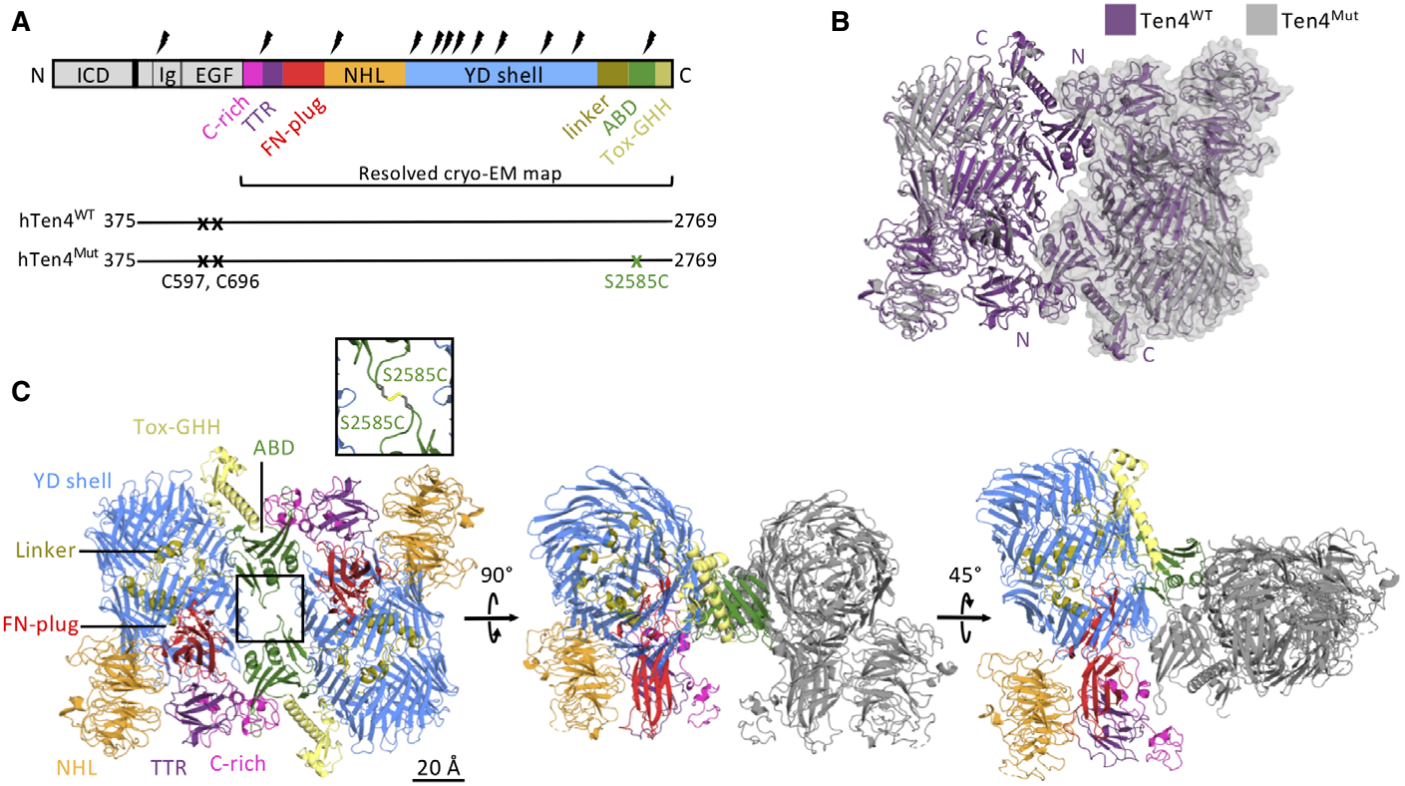
Human Teneurin4 ectodomain adopts a compact‐dimer structure Domain composition, including the covalently dimerizing cysteines in EGF repeats 2 and 5 (C597 and C696). The S2585C mutation (Teneurin4^Mut^) was introduced to stabilize the compact dimer conformation (in green). Predicted glycans are indicated as thunderbolts. Regions in grey are not represented in the cryo‐EM structure. ICD, intracellular domain; Ig, immunoglobulin fold; EGF, epidermal growth factor repeat domain; C‐rich, cysteine‐rich region; TTR, transthyretin‐related; FN‐plug, fibronectin plug; NHL, NCL, HT2A and Lin‐41; YD, tyrosine‐aspartate; ABD, antibiotic‐binding domain; Tox‐GHH, toxin‐glycine‐histidine‐histidine.Overlay of cartoon representations of human Teneurin4^WT^ (purple) and human Teneurin4^Mut^ (grey) with one chain in transparent surface to indicate the two dimer subunits. The S2585C mutation does not influence the structure of the compact Teneurin4 dimer. N and C indicate the N‐ and C‐termini, respectively.Cartoon representations of human Teneurin4^Mut^ colored by domain as indicated in (A) with the S2585C mutation, introducing an intermolecular disulfide bond, as indicated in the inset. Scale bar, 20 Å.

**Figure EV1:**
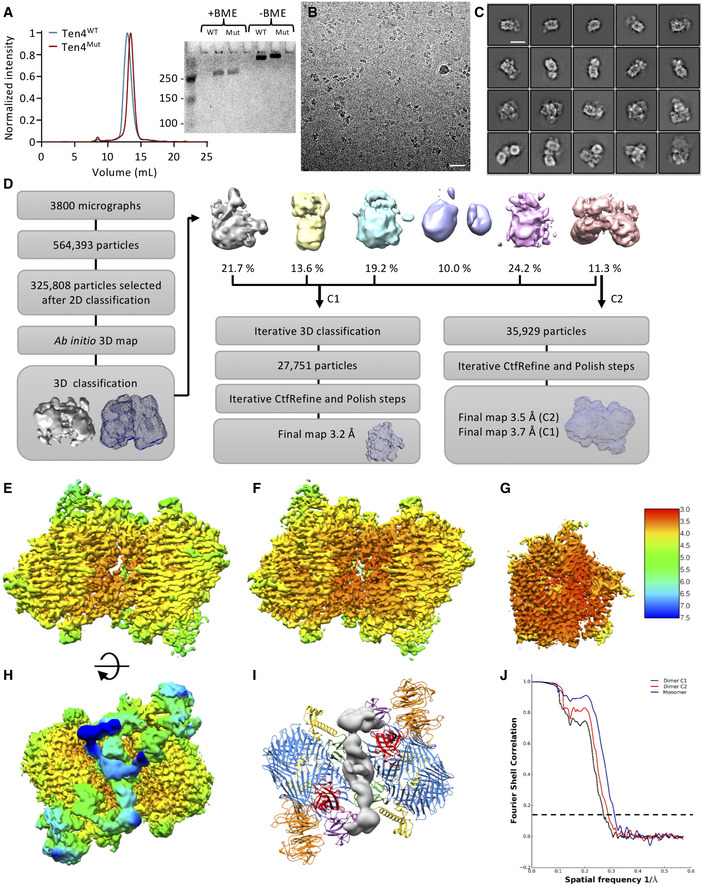
Related to Fig 1. Image reconstruction of Teneurin4^WT^ Size‐exclusion chromatography traces of purified Teneurin4^WT^ dimer (blue) and Teneurin4^Mut^ dimer (red). The difference in peak retention volume may indicate a size difference of the two Teneurin4 samples, with a smaller (or more compact) size for the Teneurin4^Mut^ dimer. Inset shows Coomassie‐blue‐stained SDS‐Page gel in presence and absence of β‐mercaptoethanol (BME) and confirms both samples are covalent dimers.Representative electron micrograph of Teneurin4^WT^. Scale bar, 40 nm.Selected 2D classes of Teneurin4^WT^. Scale bar, 10 nm.Workflow for Teneurin4 reconstruction. Note that a small subset of particles represents compact dimers, and a large subset represents extended dimers that have different conformations resulting in the reconstruction of a single subunit only.C1 symmetry reconstruction of Teneurin4^WT^ compact dimer colored by local resolution as in (G).C2 symmetry reconstruction of Teneurin4^WT^ compact dimer colored by local resolution as in (G).Reconstruction of Teneurin4^WT^ core colored according to local resolution.Same as (E), but rotated by 180° over the x‐axis.Structure of Teneurin4^WT^ in cartoon representation with EGF repeat 6–8 depicted as density maps.Fourier Shell Correlation of structures shown in E–G. Dotted line represents FSC = 0.143.

To determine the structure of covalently linked Teneurin dimers, we expressed the complete ectodomain of human Teneurin4 in HEK293 cells (Fig 1A). The protein was purified using affinity purification followed by size‐exclusion chromatography (Fig EV1A) and a 3.5 Å structure of the covalent homodimer was determined with single‐particle cryo‐electron microscopy (Figs 1B, EV1B–J, and Table 1), with the core, that is, FN‐plug, YD‐shell, linker, ABD, and Tox‐GHH combination, resolved to 3.2 Å (Fig EV1G and J). The structure revealed a novel compact conformation of the dimeric molecule. In order to obtain high‐resolution information, we inserted a stabilizing disulfide bond between the two ABD domains (S2585C, Teneurin4^Mut^) located in the core of the homodimer (Fig 1A and C inset).

**Table 1:** Cryo‐EM data collection and refinement statistics.

	Ten4^WT^ ^core focused^	Ten4^WT^ ^C1^	Ten4^WT^ ^C2^	Ten4^Mut^ ^C2^	Ten4^Mut^ ^single subunit^	Ten4^Mut^ ^TTR – C‐rich focussed^
	EMD‐12122	EMD‐12123	EMD‐12124	EMD‐12125	EMD‐12126	EMD‐12127
			7BAM	7BAN	7BAO	
Data collection and processing						
Magnification	75,000×	75,000×	75,000×	165,000×	165,000×	165,000×
Voltage (kV)	300	300	300	300	300	300
Electron exposure e^‐^/Å^2^	50	50	50	50.6	50.6	50.6
Defocus range (µm)	−2.8 to −1.1	−2.8 to −1.1	−2.8 to −1.1	−1.75 to −0.75	−1.75 to −0.75	−1.75 to −0.75
Pixel size (Å)	0.878	0.878	0.878	0.842	0.842	0.842
Symmetry imposed	C1	C1	C2	C2	C1	C1
Initial particle images (*n*)	564,393	564,393	564,393	609,582	609,582	609,582
Final particle images (*n*)	27,751	35,929	35,929	242,300	212,571	188,064
Map resolution (Å)	3.2	3.7	3.5	2.7	2.7	3.4
FSC threshold	0.143	0.143	0.143	0.143	0.143	0.143
Map resolution range (Å)	3.2–4.6	3.6–7.0	3.4–6.2	2.6–4.0	2.7–3.7	3.2–4.4
Refinement						
Initial model used (PDB code)			6FB3	6FB3	6FB3	
Model resolution (Å)			3.5	2.8	2.8	
FSC threshold			0.5	0.5	0.5	
Model composition						
Non‐hydrogen atoms			30612	30612	15306	
Protein residues			3808	3808	1904	
Ligands (glycans & Ca^2+^)			36	36	18	
B factors (Å^2^)						
Protein			73.1	66.4	64.8	
Ligand			87.6	70.9	73.0	
R.m.s. deviations						
Bond lengths (Å)			0.007	0.004	0.011	
Bond angles (°)			0.77	0.78	0.982	
Validation						
MolProbity score			2.26	1.82	1.83	
Clash score			24.0	7.9	8.0	
Poor rotamers (%)			0.36	0.18	0.18	
Ramachandran plot						
Favored (%)			94.2	94.2	94.1	
Allowed (%)			5.8	5.8	5.9	
Disallowed (%)			0	0	0	

**Figure EV2:**
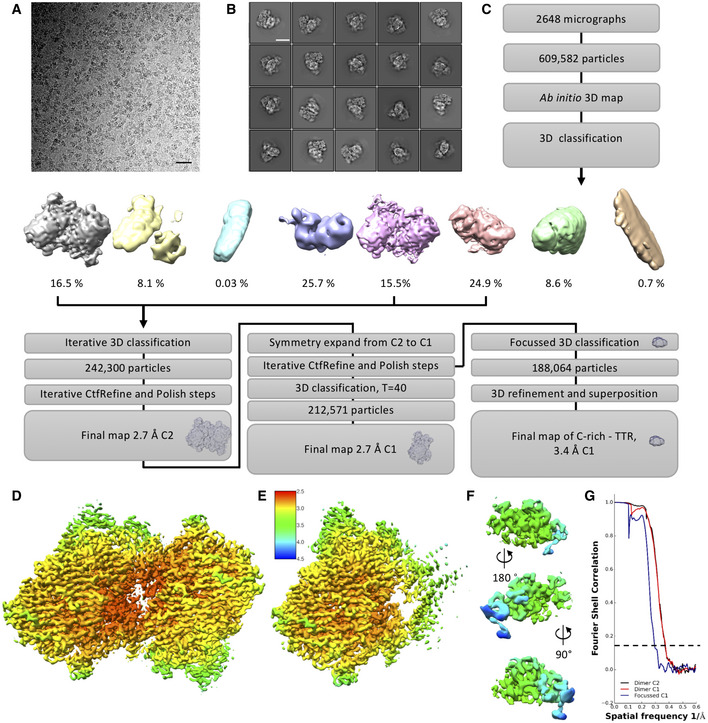
Related to Fig 1. Image reconstruction of Teneurin4^Mut^ Representative electron micrograph of Teneurin4^Mut^. Scale bar, 40 nm.Selected 2D classes of Teneurin4^Mut^. Scale bar, 10 nm.Workflow for Teneurin4^Mut^ reconstruction.C2 symmetry reconstruction of Teneurin4^Mut^ colored by local resolution. Coloring as in (E).Symmetry expanded half‐dimer reconstruction of Teneurin4^Mut^ colored by local resolution. The map quality of the C‐rich region, TTR, FN‐plug and NHL domains is improved in this reconstruction.Three different side views of a focused reconstruction of Teneurin4^Mut^ C‐rich–TTR. Top panel same orientation as D and E.Fourier Shell Correlation of structures shown in D–F. Dotted line represents FSC = 0.143.

This provided a 2.7 Å reconstruction of the compact dimer conformation (Figs 1B and C, and EV2A–G). Alignment of the dimeric Teneurin4^WT^ and Teneurin4^Mut^ structures indicates that the structure of Teneurin4^Mut^ is virtually identical to the Teneurin4^WT^ protein (root mean square deviation (r.m.s.d.) = 0.5 Å for all 3,808 Cα atoms, Fig 1B). Dimensions of the compact dimer are 166 Å by 133 Å by 112 Å. A total of 12 predicted glycans on the extracellular domain (ECD) are expected to expand these dimensions (Figs 1A and EV5C); however, they are not fully resolved in our cryo‐EM maps due to their flexible nature. A two‐fold symmetry axis of the structure is positioned between the loops of the ABD domains (I2584–N2588) and the beta‐strands of the YD‐shells (L1660–S1686). The C1 reconstruction of the Teneurin4^WT^ dimer reveals that EGF repeats 6–8 are positioned close to the YD shell and ABD domains, and that EGF8 links to a previously unresolved domain containing a cysteine‐rich region that we denote C‐rich domain (Fig EV1H and I). The ECD segment preceding EGF6 is not resolved in the reconstructed map and EGF repeats 6–8 are of considerably lower resolution (> 5 Å) compared to the rest of the ECD region (Fig EV1H). It is thus unclear if the EGF repeats have direct contacts with the YD shell and ABD domains in the dimer. Conceivably, the EGF repeat domain as a whole, that harbors the conserved intermolecular disulfide bonds in EGF2 and EGF5, contributes to stabilizing the compact dimer composition.

**Figure 2:**
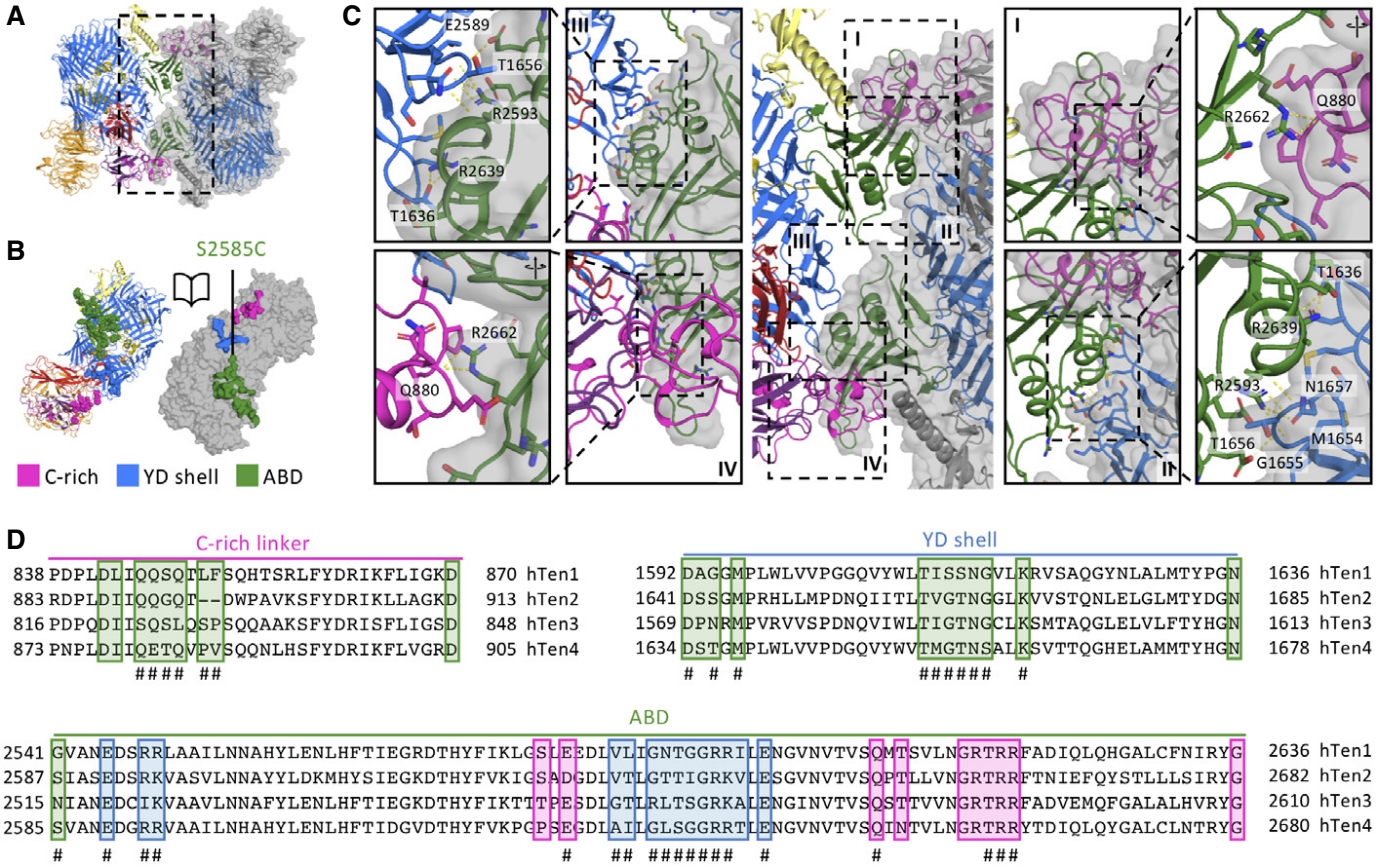
Dimer interface of human Teneurin4 Cartoon representation of the Ten4^Mut^ dimer, with one subunit also shown as transparent surface model. Dashed rectangle indicates area shown in (C) (central panel). Domains are colored as in Fig 1A.Open book representation and surface model of domains involved in the dimer interface. Residues involved in the dimer interface are shown as spheres. Colors of the domains are shown as in Fig 1A.The ABD domain (green) contacts the YD‐shell (blue) and the C‐rich region (pink). Central panel depicts overview of the dimer interface. Roman numbers in mid‐panels refer to location in central panel. Outer panels are rotated and zoom ins for better visualization of interacting residues. Hydrogen bonds are indicated by yellow dashed lines.Sequence alignment of all four human Teneurins. Residues that are part of the dimer interface are highlighted in the color of the interacting domain of the other monomer, and # represents the residues buried in the dimer interface by a surface area higher than 5 Å^2^.

The compact dimer interface is formed by interactions of the ABD domain with the YD‐shell and C‐rich region of the other monomer (Fig 2A and B). Specifically, residues R2593, E2589, and R2639 of the ABD domain interact with two loops of the YD shell (T1636 in the loop spanning residues 1634–1638 and M1654‐G1655‐T1656 in the adjacent loop spanning residue 1,654–1,659, Fig 2Cpanel II and III), while R2662 of the ABD domain interacts with Q880 of the C‐rich region (Fig 2C panels I and IV). The 2472 Å^2^ buried surface area in the dimer interface is predominantly hydrophilic with only very few hydrophobic contacts. The ABD domain is central in the interface and contributes most to the buried surface area (Table 2, Fig 2D). Notably, the potential furin cleavage site RTRR (Vysokov *et al*, 2016) (amino acids 2,659–2,662 in human Teneurin4) in the ABD domain is intact and partially buried in the dimer interface, precluding enzymatic processing in this compact conformation.

**Table 2:** Domain‐specific buried surface area (BSA) in Å^2^.

	Total BSA (Å²)
C‐rich	234
TTR	0
FN‐plug	0
NHL	0
YD shell	378
Linker	0
ABD	623
Tox‐GHH	0

**Figure 3:**
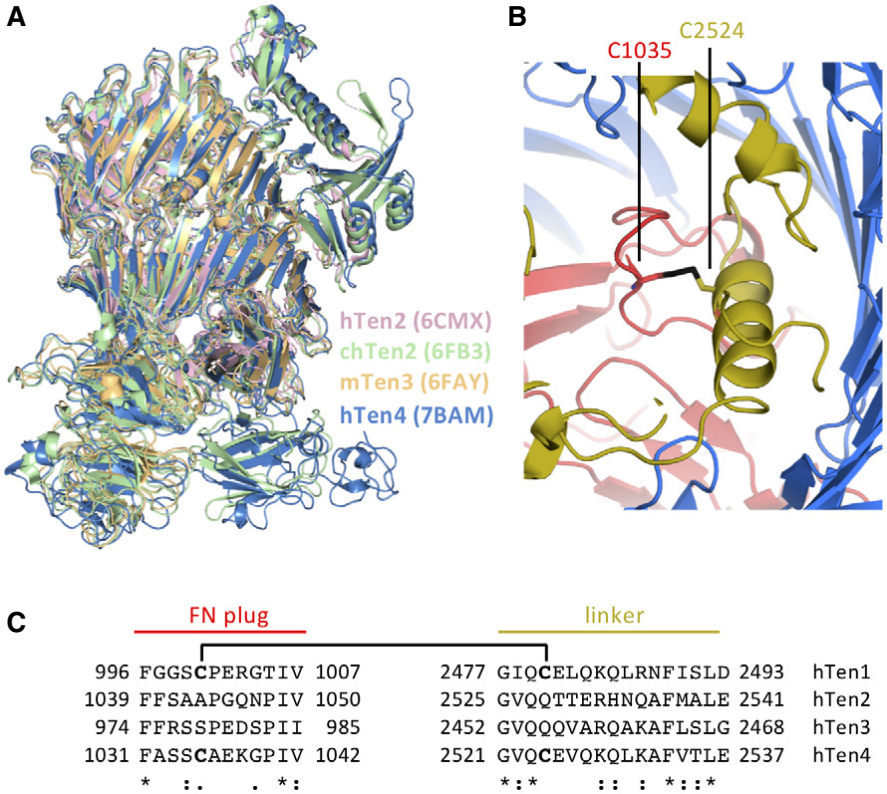
Structures of Teneurins are similar Overlay of cartoon representations of human Teneurin2 (6CMX) (Li *et al*, 2018), chick Teneurin2 (6FB3) (Jackson *et al*, 2018), mouse Teneurin3 (6FAY) (Jackson *et al*, 2018) and human Teneurin4 (7BAM; this paper). R.m.s.d 1.8 Å (over 1735 Cα atoms) for chick Teneurin 2 and human Teneurin4, 2.0 Å (over 1230 Cα atoms) for human Teneurin2 and human Teneurin4 and 1.9 Å (over 1431 Cα atoms) for mouse Teneurin3 and human Teneurin4.Cartoon representation of the intramolecular disulfide bond between C1035 of the FN‐plug domain (red) and C2524 of the linker region (yellow) in human Teneurin4.Sequence alignment of the FN‐plug—linker cysteines and flanking residues. The interdomain disulfide‐forming cysteines are in bold and indicated by a bracket. The consensus symbols are according to Clustal Omega.

**Figure EV3:**
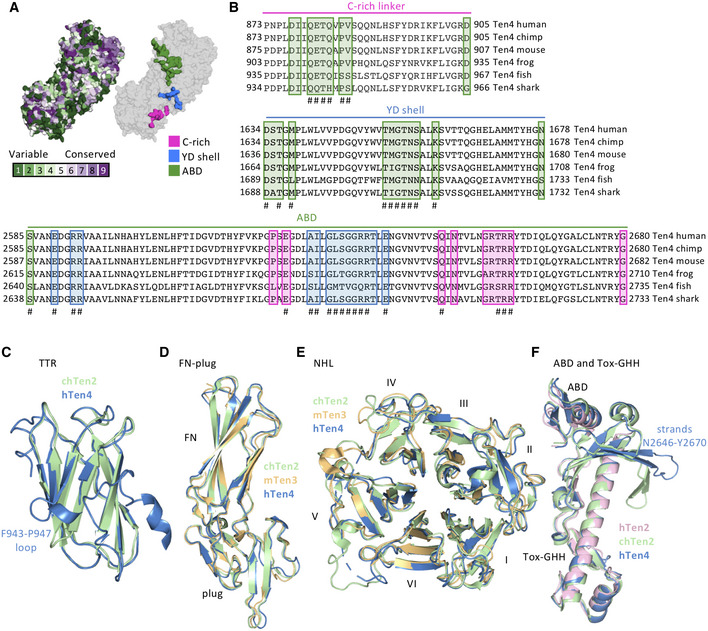
Related to Figs 2 and 3. Domain‐specific comparisons of published Teneurin structures Surface representation of evolutionary conserved score (left panel) and dimer interface residues (right panel), colored as in Fig 1A.Amino acid alignment of interface residues in vertebrates. Coloring as in Fig 2D.Overlay of cartoon representations of the TTR domain of chick Teneurin2 (6FB3; green) and human Teneurin4 (7BAM; blue), r.m.s.d. 1.0 Å for 80 Cα atoms.Overlay of cartoon representations of the FN‐plug domain of chick Teneurin2 (6FB3; green), mouse Teneurin3 (6FAY; orange) and human Teneurin4 (7BAM; blue), r.m.s.d. 0.8 Å over 185 Cα atoms for chick Teneurin2 and human Teneurin4, and 1.4 Å over 185 Cα atoms for mouse Teneurin3 and human Teneurin 4.Overlay of cartoon representations of the NHL domain of chick Teneurin2 (6FB3; green), mouse Teneurin3 (6FAY; orange) and human Teneurin4 (7BAM; blue). Highest r.m.s.d. 1.2 Å over 326 Cα atoms for mouse Teneurin3 and human Teneurin 4.Overlay of cartoon representations of the ABD and Tox‐GHH domains of human Teneurin2 (6CMX; pink), chick Teneurin2 (6FB3; green) and human Teneurin4 (blue). R.m.s.d. of both domains equals 1.0 Å over 135 Cα atoms for human Teneurin2 and human Teneurin4, and 0.8 Å over 189 Cα atoms for chick Teneurin2 and human Teneurin4.

A structural comparison with all three published Teneurin ECD monomer subunits (Jackson *et al*, 2018; Li *et al*, 2018) reveals a striking resemblance (Fig 3A), with r.m.s.d. scores ranging from 1.8 Å to 2.0 Å. Specifically, the r.m.s.d. score for hTen2 vs hTen4 is 2.0 Å for 1,230 Cα atoms; for chTen2 vs hTen4, 1.8 Å for 1,735 Cα atoms; and for mTen3 vs hTen4, 1.9 Å for 1,431 Cα atoms. This similarity is also observed at the single domain level (Fig EV3C–F), exposing only two minor structural differences. First of all, we observed an extra disulfide bond between C1035 of the FN‐plug and C2524 of the linker domain, covalently locking the core domains into the so‐called superfold (Fig 3B). Based on sequence analysis, this disulfide bond is also present in human Teneurin1, but not in human Teneurin2 or human Teneurin3 (Fig 3C). Furthermore, the arrangement of two beta‐strands (N2646–Y2670) in the ABD domain of Teneurin4 is deviating from the other Teneurins (Fig EV3F). This could be explained by the compact dimer arrangement of Teneurin4, where this specific loop is part of the dimer interface, while the structures of all other Teneurins were determined from monomerized samples. The overall high structural similarity of the family members, in combination with the nonoverlapping expression patterns as described previously (Ben‐Zur *et al*, 2000; Zhou *et al*, 2003), suggests a high level of functional redundancy. Indeed, *in vitro* binding studies of Teneurins and Latrophilins—where context plays no role—reveal substantial promiscuity (Burbach & Meijer, 2019). Altogether, our cryo‐EM density maps reveal a novel compact conformation of covalently dimerized Teneurin4, while maintaining the overall structure of the superfolds.

### Cysteine‐rich domain structure reveals three calcium‐binding sites

**Figure 4:**
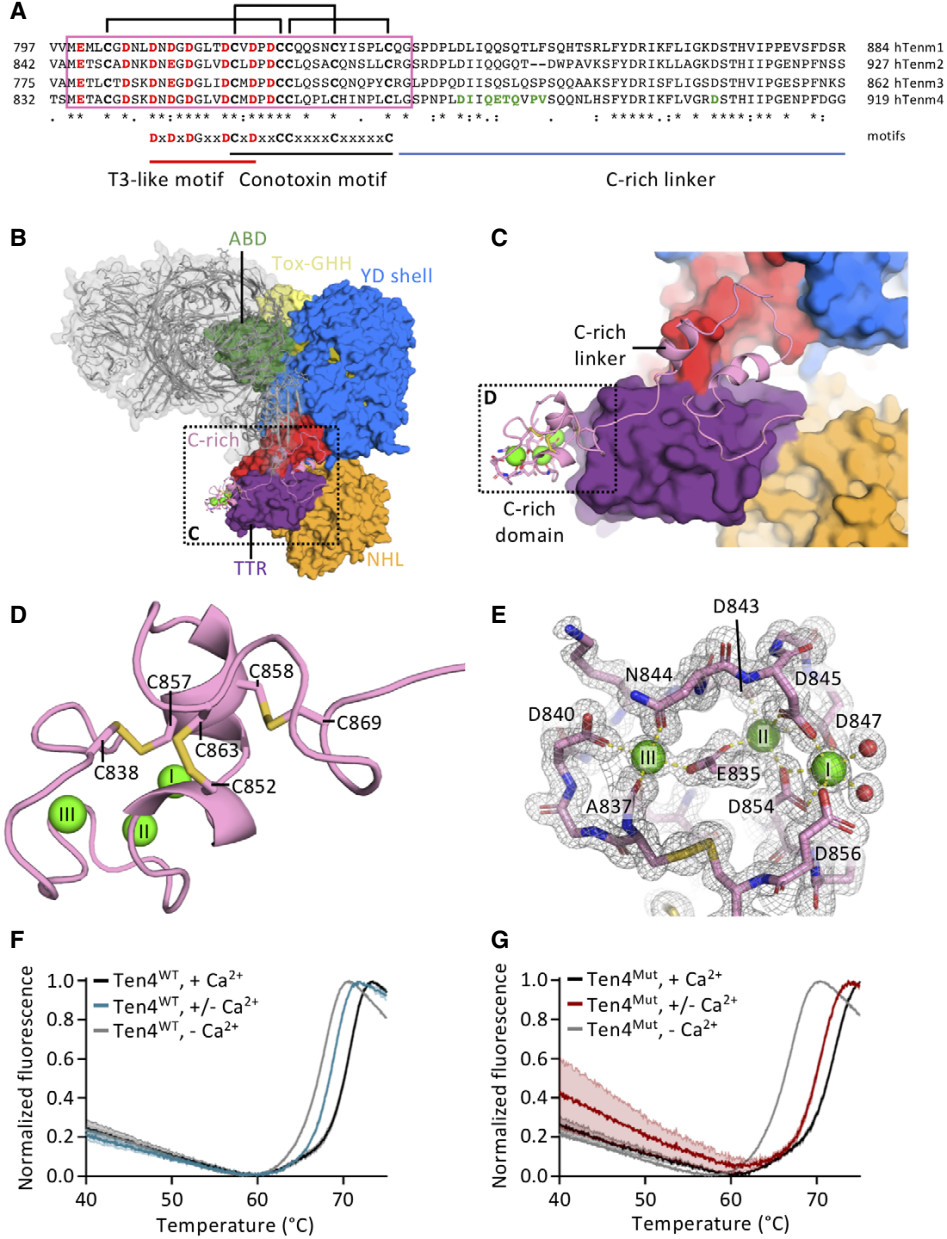
The C‐rich region is a calcium‐binding domain and linker ASequence alignment of the C‐rich region, including disulfide bonds as brackets, calcium‐coordinating acidic residues in red and domain interface residues in green. The consensus symbols are according to Clustal Omega. Pink box highlights the C‐rich domain in the four human Teneurins.BThe Teneurin4 dimer with one chain colored grey in cartoon and transparent surface representation, and the other chain colored according to Fig 1C with the C‐rich domain and C‐rich linker in cartoon representation and all other domains in surface representation. Region shown in C indicated with dashed box.CThe C‐rich domain and C‐rich linker straddle the TTR domain, colored purple in surface representation. The calcium ions are shown in green. Coloring as in B. C‐rich domain shown in D indicated with dashed box.DC‐rich domain in cartoon representation with disulfide bonds in stick representation and labeled. Calcium ions are represented by green spheres.EStick representation and 2mF_obs_‐DF_calc_ electron density map at 1σ level of the C‐rich domain showing that three calcium ions (I–III) are each ligated in octahedral coordination. Calcium‐coordinating residues are labeled.F, GThermal shift assay traces of human Teneurin4^WT^ (F) and human Teneurin4^Mut^ (G) in the presence of physiological calcium concentrations (+ Ca^2+^; black), low calcium concentration (+/− Ca^2+^; blue and red, respectively) and no calcium (− Ca^2+^; grey). Data information: Data are represented as mean ± SEM. Each condition was performed in triplicates.

**Figure EV4:**
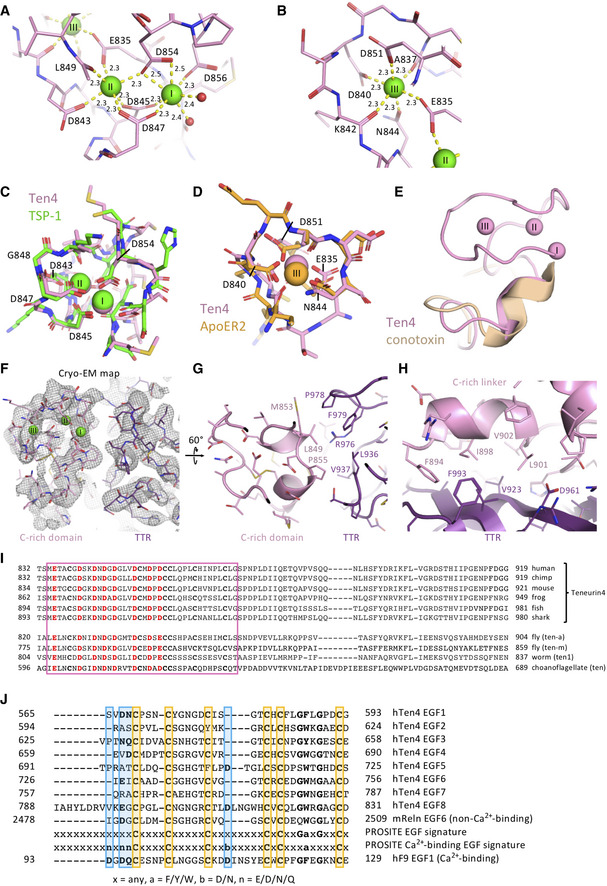
Related to Fig 4. C‐rich domain calcium coordination and homology A,BOctahedral coordination of calcium ions (A) I, II, and (B) III by (mostly) negatively charged residues or backbone carbonyl oxygens of labeled residues. Calcium‐coordinating bond distances are indicated in Å and consistent with expected calcium coordination.CCoordination of two calcium ions (I and II) in the C‐rich domain of Teneurin4 (pink) matches with the coordination of two calcium ions in thrombospondin type 3 (T3) repeats (green, Thrombospondin 1 ‐ TSP‐1; PDB 1UX6; Kvansakul *et al*, 2004).DCoordination of the third calcium ion (labeled III) in the Teneurin4^WT^ C‐rich domain matches with the coordination of the calcium ion in the low‐density lipoprotein receptor (LDLR) class A (LA1) modules (orange, Apolipoprotein E receptor 2‐ApoER2; PDB 3A7Q; Yasui *et al*, 2010).EPart of the C‐terminal side of the C‐rich domain (residues 855‐864) has structural similarity to conotoxins (light brown, A‐family conotoxin; PDB 7EDK). The calcium ions (I‐III) in the Teneurin4^WT^ C‐rich domain are shown for reference.FCryo‐EM density map (see also EV2F) of the C‐rich domain and TTR region. Orientation of the C‐rich domain is similar as in Fig 4E.GIntramolecular C‐rich domain–TTR interaction. Residues at the interface are labeled.HIntramolecular C‐rich linker–TTR interaction. Residues at the interface are labeled.IDisulfide bond forming cysteine residues (bold and black) and calcium coordinating acidic residues (bold and red) are fully conserved among Teneurins in the animal kingdom ranging from choanoflagellate and worm through to mammals.JNone of the Teneurin4 EGFs contain the canonical calcium‐binding EGF signature sequence. Sequence alignment of human Teneurin4 EGF domains, compared to a non‐calcium‐binding EGF domain from mouse Reelin (UNIPROT: Q60841) and a calcium‐binding EGF domain from human Factor IX (UNIPROT: P00740), as well as the regular and calcium‐binding EGF signature as defined by PROSITE. Residues directly involved in calcium‐binding are indicated in blue, while cysteines are indicated in yellow. Residues that agree with the signatures are indicated in bold. Lack of the calcium binding signature in all of the human Teneurin4 EGFs may indicate that the Teneurin4 EGFs are not involved in calcium binding although a role for the EGFs in calcium binding cannot be fully excluded.

Using a combination of XRD and cryo‐EM, we determined the structure of the previously unresolved EGF8—TTR domain‐linking segment (residues 834–919), that includes the highly conserved C‐rich region (Figs 4A and EV4I; Tucker *et al*, 2012). The cryo‐EM map reveals a compact domain (residues 834–871, denoted C‐rich domain) (Fig EV2C, F and G) at the N‐terminal side of this linker that contains eight acid amino acids and six cysteines. We determined a 1.5 Å resolution crystal structure of this Teneurin4 C‐rich domain (Fig 4B–E, Table 3). In the C‐rich domain, three calcium ions are fully coordinated, each in octahedral geometry, by side chains of the eight conserved acidic residues (E835, D840, D843, D845, D847, D851, D854, and D856), as well as N844, the backbone carbonyl oxygens of A837, K842, and L849, and two water molecules (Figs 4E and EV4A and B). The coordination of two calcium ions (I and II in Fig 4D and E) is similar to Thrombospondin type 3 repeats (T3) with a matching calcium‐binding motif (Kvansakul *et al*, 2004; Figs 4A and EV4C) and the coordination of the third calcium resembles that of LDL receptor type‐A (LA) modules (Blacklow, 2007; Yasui *et al*, 2010; Fig EV4D). The six cysteines in the C‐rich domain form three disulfide bonds in the pattern 1–3, 2–5, 4–6 (C838–C857, C852–C863, and C858–C869, Fig 4D) also observed in LA modules (Yasui *et al*, 2010). The C‐terminal side of the C‐rich domain (residues 857–869) is structurally similar to disulfide‐bond rich toxic peptides from the marine cone snail, known as conotoxins (Figs 4A and EV4E). Despite the partial similarities, the Teneurin4 C‐rich domain as a whole does not structurally resemble other known domain folds. The C‐rich domain is connected to the TTR domain by an alpha‐helix containing linker (residues 872–919) that wraps around the TTR domain (Figs 4B and C, and EV4F). The C‐rich domain and the alpha‐helix containing linker have mixed hydrophobic and hydrophilic interactions with the TTR domain (Fig EV4G and H).

**Table 3:** Crystallographic table.

	Native	Anomalous
	7PLP	
Data collection and processing		
Space group	*P* 2_1_ 3	*P* 2_1_ 3
a, b, c (Å)	66.34	66.69
α, β, γ (deg)	90.0	90.0
Wavelength (Å)	0.7749	1.7712
Resolution (Å)	46.91–1.50 (1.53–1.50)^a^	47.16–1.86 (1.90–1.86)^a^
Rmerge	0.164 (2.503)	0.143 (1.028)
Rpim	0.026 (0.399)	0.034 (0.663)
CC(1/2)	0.999 (0.891)	0.998 (0.416)
No. of observations	660,948 (30,971)	244,883 (1,583)
No. unique	15,908 (769)	8,169 (315)
Mean I/σ (I)	16.9 (2.0)	17.0 (1.5)
Completeness (%)	100 (100)	95.7 (63.7)
Redundancy	41.5 (40.3)	30.0 (5.0)
Data processing		
Rwork/Rfree	0.134/0.151	
Model composition		
Nonhydrogen atoms	710	
Protein residues	83	
Ligands	13	
Water	58	
B factors (Å^2^)		
Protein	23.93	
Ligand	36.34	
R.m.s. deviations		
Bond lengths (Å)	0.0151	
Bond angles (°)	1.89	
Validation		
MolProbity score	1.21	
Clash score	3.28	
Poor rotamers (%)	1.30	
Ramachandran plot		
Favored (%)	94.94	
Allowed (%)	5.06	
Disallowed (%)	0.0	

^a^ Values in parenthesis are for the highest resolution shell.

To investigate the effects of calcium on protein stability, we determined the thermal unfolding of Teneurin4^WT^ and Teneurin4^Mut^ using SYPRO Orange. The melting temperature (T_m_) of Teneurin4^WT^ in the presence of 2 mM Ca^2+^ (physiological calcium, + Ca^2+^) was approximately 3°C higher compared to a condition where Ca^2+^ was removed by buffer exchange and 2‐mM divalent metal‐ion chelator EDTA was added to remove any remaining protein‐bound calcium (no calcium, − Ca^2+^, Fig 4F). The melting temperature of Teneurin4^WT^ in the condition where excess calcium was removed by SEC buffer exchange (low calcium, +/− Ca^2+^) showed an increase of 1°C compared to no calcium, placing it in between the physiological and no calcium conditions (Fig 4F). Teneurin4^Mut^ was also destabilized upon removing calcium, with T_m_ shifts of approximately 1°C and 5°C compared to low and no calcium conditions, respectively (Fig 4G). Sequence analysis indicates that none of the predicted EGF‐repeats in Teneurin4 are classified as having the EGF calcium‐binding signature (Fig EV4J); however, we cannot exclude that calcium binding to the EGF repeats contributes to the observed shift in thermostability. The resolved structure of the calcium‐binding C‐rich domain, together with the thermostability data, highlights that calcium binding plays a role in the stabilization of Teneurin4, and although calcium is not directly involved in dimer interactions, it may contribute to the observed compactness of the dimeric molecule.

### In solution structure determination confirms compact conformation of Teneurin4

**Figure 5:**
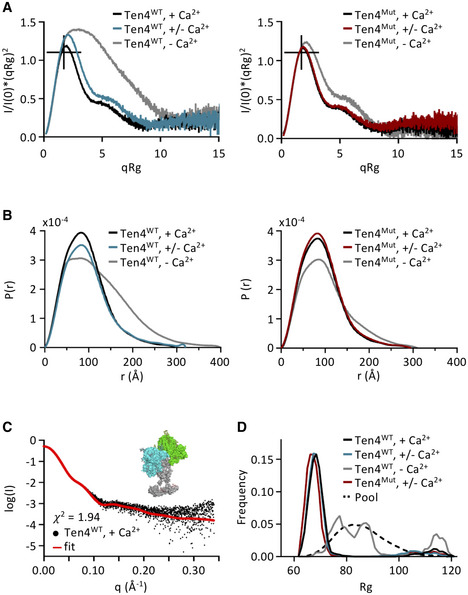
Teneurin4^WT^ adopts the compact dimer conformation in the presence of calcium Dimensionless Kratky plots for Teneurin4^WT^ (left) and Teneurin4^Mut^ (right) in physiological calcium concentrations (+ Ca^2+^; black), low calcium concentrations (+/− Ca^2+^; blue and red, respectively) and no calcium (− Ca^2+^; grey). I(0) and Rg were obtained from Guinier analysis (Fig EV5A and B). Crosshairs indicate the maximum position for a fully folded globular protein.Pair‐distance distribution plots for Teneurin4 proteins colored as in A.Full‐length Teneurin4^WT^ ECD model (inset) and fit (red line) against the Teneurin4^WT^ data in physiological calcium concentrations (black dots).Distributions of radii of gyration of ensembles of structures selected from the random pool (dotted line), to best represent the SAXS data of each of the Teneurin4 variants, colored as in A.

**Figure EV5:**
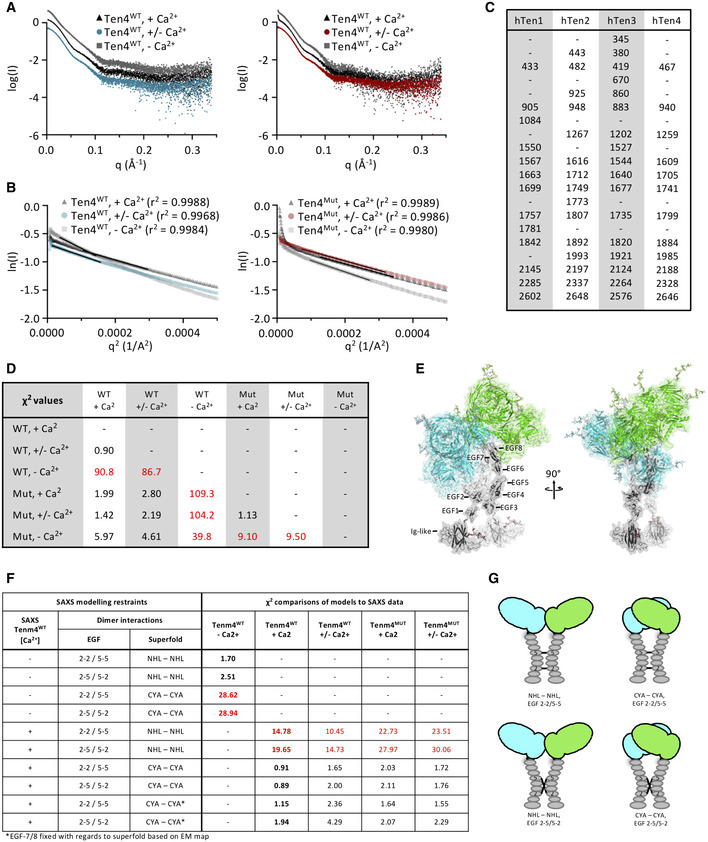
Related to Fig 5. Teneurin4^WT^ and Teneurin4^Mut^ are compact *cis* dimers in the presence of physiological calcium ASAXS Log I versus q plots for Teneurin4^WT^ (left) and Teneurin4^Mut^ (right) in physiological calcium concentrations (+ Ca^2+^; black), low calcium concentrations (+/− Ca^2+^; blue and red, respectively) and no calcium (− Ca^2+^; grey).BSAXS‐based Guinier plots for Teneurin4 proteins colored as in A.CTable indicating predicted sugars positions according to the NetNGlyc server for human Teneurin proteins (Uniprot codes Teneurin1, Q9UKZ4; Teneurin2, Q9NT68; Teneurin3, Q9P273; Teneurin4, Q6N022).DTable indicating the χ^2^ values of the SAXS data comparison of Teneurin4^WT^ and Teneurin4^Mut^ ECD in different calcium conditions.ECartoon representation of the best‐fitting SAXS‐based rigid‐body model, same as in Fig 5C, inset. The two different superfolds in the covalent dimer are colored green and cyan, respectively. The Ig‐like and EFG domains, placed by SAXS‐based rigid body modeling, are colored light and dark grey to indicated the two chains in the dimer. Modeled glycans are in stick representation. Modeling parameters for this model: Ten4^WT^, + Ca^2+^, 2‐5/5‐2 EGFs, CYA‐CYA interface.F–GModels of the full Teneurin4 ectodomain were generated using CORAL (Petoukhov *et al*, 2012). The CORAL‐based rigid body models inform on how well the models fit (or do not fit) the SAXS data. The EGF and Ig domain stalk, colored grey in panel E and Fig 8, represents the best fitting conformation but other conformations of the EGF‐Ig stalk also fit the data. The scattering data of the Teneurin4^WT^ protein in either no calcium or physiological calcium concentration were used for modelling, also indicated in the comparison table in bold. To model *cis* dimers, interactions were enforced between the EGFs and superfolds of the intermolecular chains using distance restraints, indicated in cartoons in G. For the EGFs, inter‐dimer covalent disulfide bonds were restrained either from EGF2 to EGF2 and EGF5 to EGF5 from one chain to the other as in a “parallel” fashion, or from EGF2 to EGF5 and EGF5 to EGF2, as in an “anti‐parallel” fashion. Superfolds of opposite chains were restrained to form the C‐rich/YD shell/ABD interface (CYA; this paper), or a previously described interface (NHL‐NHL interface), involving a splice site in the NHL domain, resulting in more elongated particles (Jackson *et al*, 2018; Li *et al*, 2020). In addition, the effect was assessed of fixing the position of EGF7 and EGF8 with regard to the superfold, based on the low‐thresholded C1 EM map (indicated by a star). χ^2^ comparison of models to data, as calculated by CRYSOL (Svergun *et al*, 1995), are indicated at the right side of the table. There was no substantial difference in comparing model to data for the two EGF *cis*‐dimerization modes. In addition, models obtained by fixing the positions of EGF‐7 and 8 with regard to the Teneurin4 superfold, compared to not fixing those positions, explain the scattering data at physiological calcium concentrations equally well, again indicating that the SAXS and cryo‐EM data in the presence of calcium are very similar. *Trans* models, in which two Teneurin4 *cis* dimers interact, do not fit to the SAXS data as the rigid body modelling leads to severe steric classes.

In order to characterize the conformation of Teneurin4^WT^ and Teneurin4^Mut^ in solution, we performed small angle x‐ray scattering (SAXS) measurements. Similar to the thermostability experiments, we compared SAXS signal in physiological, low and no calcium conditions for both purified proteins (Figs 5A and EV5A, and Table 4). Sample quality and parameters were determined using Guinier analysis (Fig EV5B). Datasets of both Teneurin4 variants collected at physiological and low calcium conditions are similar. They correlate with χ^2^ values that range from 0.90 to 2.80 (Fig EV5D), indicating structural similarity in solution between the wildtype and the mutant Teneurin4 that has the compact dimer interface enforced by the S2585C mutation. From the dimensionless Kratky plots, it becomes clear that Teneurin4 in these conditions displays a typical multidomain protein curve with a peak close to the theoretical maximum for a globular protein (Fig 5A). Likewise, the pair‐distance distribution indicates that both Teneurin4 variants are of a mostly globular nature (Fig 5B). This suggests that the majority of Teneurin4^WT^ molecules in solution are in a compact dimer conformation in the presence of calcium (physiological and low concentration), similar to that enforced by the mutant.

**Table 4:** SAXS data overview and analysis.

Construct	Buffer	Rg (Å)	Dmax (Å)	Porod volume (nm^3^)	SASBDB code
Ten4^WT^	+/− Ca^2+^	78.09 ± 0.24	320	1,291	SASDKT4
Ten4^WT^	+ Ca^2+^	71.90 ± 0.14	295	1,188	SASDKS4
Ten4^WT^	− Ca^2+^	95.38 ± 0.25	400	1,263	SASDKU4
Ten4^Mut^	+/− Ca^2+^	70.34 ± 0.10	293	1,196	SASDKW4
Ten4^Mut^	+ Ca^2+^	69.88 ± 0.12	292	1,174	SASDKV4
Ten4^Mut^	− Ca^2+^	77.94 ± 0.18	310	1,274	SASDKX4

We have generated a series of restrained rigid‐body models of the Teneurin4 ECD dimer, using the SAXS data of Teneurin4^WT^ at 2 mM CaCl or 2 mM EDTA (i.e., physiological and no calcium), to probe the role of several Teneurin4 dimer interfaces by independently restraining them (Fig EV5E–G). The rigid‐body models include the two Teneurin superfolds, as well as the eight predicted EGF domains (residues 564–833) modeled based on homology, and a novel predicted Ig‐fold (residues 375–563) modeled based on interresidue contacts and distances from co‐evolutionary data. The predicted Ig‐fold spans residues V438 to E561, and contains a total number of 7 predicted beta‐strands and a ~40 residue loop between the fourth and fifth beta‐strand. Note that the SAXS data does not allow us to distinguish between a covalent EGF‐mediated *cis* dimer in “parallel” (i.e. intermolecular EGF2‐EGF2 and EGF5‐EGF5 disulfide bonds) or “anti‐parallel” fashion (i.e. intermolecular EGF2‐EGF5 disulfide bonds), as the derived models fit equally well to the SAXS data of Teneurin4 at physiological and low calcium conditions (Fig EV5F). We refer to the interface of the compact dimer as CYA‐CYA (C‐rich/YD‐shell/ABD; this paper) interface, and the interface of the recently described splice‐site dependent beta‐propeller‐mediated dimerization interface as NHL–NHL interface (Berns *et al*, 2018; Jackson *et al*, 2018; Li *et al*, 2020). Our analysis indicates that the CYA–CYA interface explains the SAXS data of Teneurin4^WT^ and Teneurin4^Mut^ in physiological and low calcium conditions (χ^2^ values of 0.89–2.11), but not that of Teneurin4^WT^ in the condition that lacks any calcium (χ^2^ values of 28.62–28.94; Figs 5C and EV5E–G). Teneurin4^WT^ in the condition that lacks calcium is more extended (Fig 5B) and structurally different from the Teneurin4 conformation in the calcium‐containing conditions (χ^2^ values of 86.7–109.3; Fig EV5D). Taken together, this may indicate that the CYA–CYA interface is abrogated in the absence of calcium. Interestingly, the Teneurin4^WT^ data in no calcium condition is explained well by a model in which the splice‐site dependent NHL–NHL interface is restrained (χ^2^ values of 1.70–2.51), whereas such an NHL‐restrained dimer model does not explain the Teneurin4 data in the presence of calcium (χ^2^ values of 10.45–30.06) (Fig EV5E–G). The SAXS data indicate that the Teneurin4 ECD can adopt different dimer conformations and that these conformations are dependent on the presence of calcium.

Finally, to validate our hypothesis that Teneurin4^WT^ has a compact dimer arrangement in solution, we performed ensemble modeling. We generated a pool of 10,407 structures using structural constrains obtained from the rigid‐body model shown in Figs 5C and EV5E, maintaining the physiological EGF dimerization interface, but introducing flexible linkers between the Ig‐fold, EGF1, EGF2‐5, EGF6, EGF7, EGF8 and the superfold subunits in the dimer (residues 834–2769), allowing them to move independently as rigid bodies. Ensembles of structures that best represent the experimental data were automatically selected. Whereas a broad ensemble of structures fits the data for Teneurin4^WT^ without calcium, narrower ensembles containing only the most compact structures fit the data for Teneurin4^WT^ in the calcium containing conditions (Fig 5D), confirming reduced flexibility and structural compaction in the presence of calcium. Together, these data indicate that Teneurin4^WT^ molecules are, predominantly, compact dimers in solution, and that calcium has a stabilizing role in maintaining this conformation.

### Teneurin4 induces filopodia formation and is localized at filopodial tips

**Figure 6:**
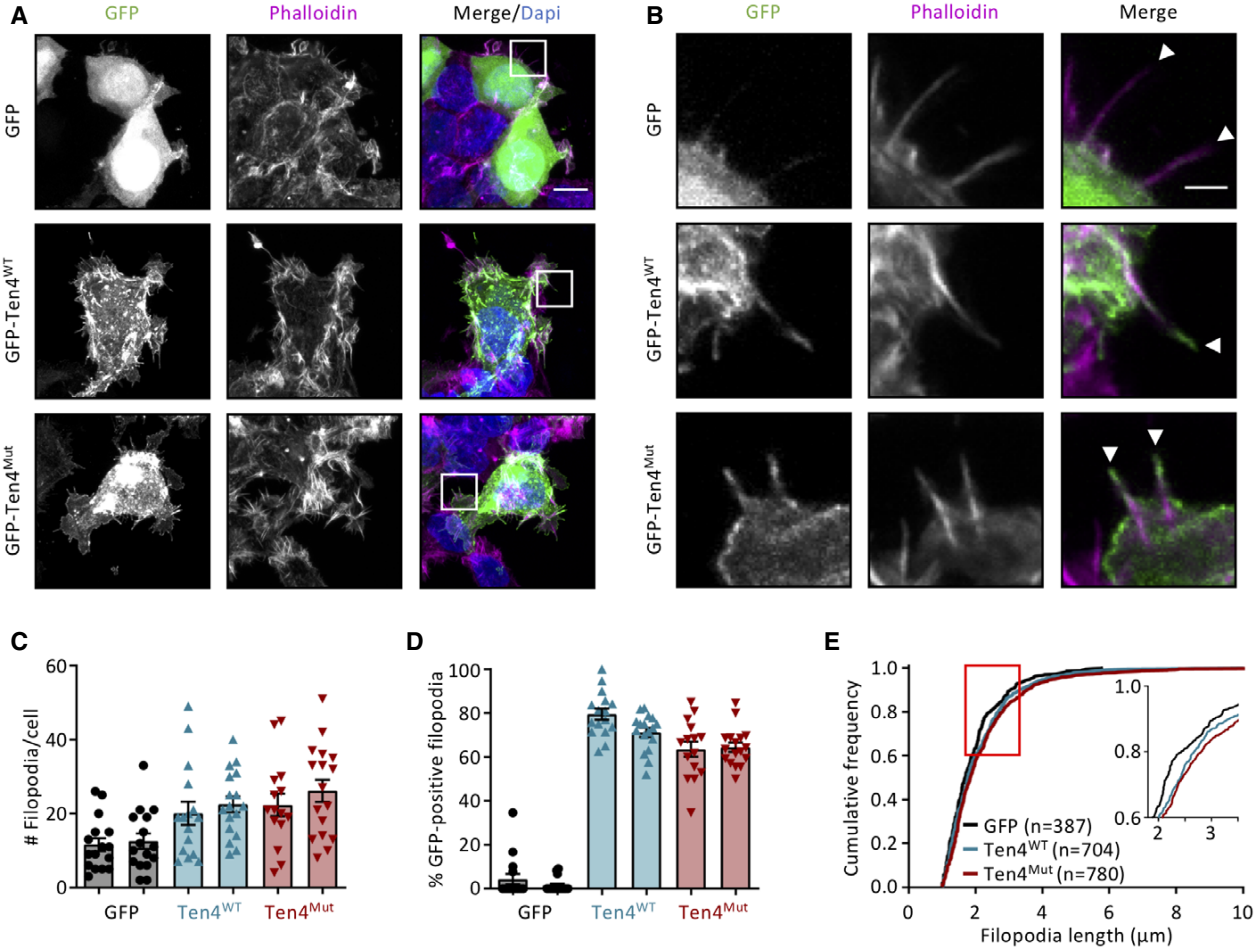
Overexpression of full‐length transmembrane Teneurin4^WT^ and Teneurin4^Mut^ induces the formation of filopodia Representative images of HEK293‐T cells transfected with GFP (top), GFP‐Teneurin4^WT^ (GFP‐Ten4^WT^; middle) or GFP‐ Teneurin4^Mut^ (GFP‐Ten4^Mut^; bottom) for 24 h and stained with Alexa 568 Phalloidin (magenta) and Dapi (blue). Images are maximum intensity projections of 14–16 stacks. Scale bar, 10 µm.Zoom shows the regions indicated in A. White arrows indicate the tip of the filopodium from the transfected cell. Scale bar, 2 µm.Average number of filopodia per cell in cells transfected with GFP, GFP‐Teneurin4^WT^ (Ten4 ^WT^; red) and GFP‐ Teneurin4^Mut^ (Ten4^Mut^; blue).Fraction of filopodia enriched with GFP per cell in GFP‐, GFP‐Teneurin4^WT^ (Ten4^WT^; blue)‐ and GFP‐Teneurin4^Mut^ (Ten4^Mut^; red)‐transfected cells.Cumulative distributions of the length of individual filopodia in cells transfected with GFP, GFP‐Teneurin4^WT^ (Ten4^WT^) and GFP‐Teneurin4^Mut^ (Ten4^Mut^). The box highlights the zoomed area. *n* indicates the number of fliopodia analyzed per condition. Data information: Data are represented as mean ± SEM. Each symbol represents an individual cell. Data from 15 to 17 cells per condition for each independent experiment.

Teneurin2 and Teneurin4 have previously been shown to induce cellular protrusions or filopodia in COS7‐A and neuroblastoma cells, respectively (Rubin *et al*, 2002; Suzuki *et al*, 2014). In order to confirm that the mutation introduced in Teneurin4 does not compromise its cellular function and localization, we transfected mammalian HEK293‐T cells with GFP‐tagged full length Teneurin4^WT^ or Teneurin4^Mut^ constructs. Both Teneurin4 constructs were expressed and localized to the membrane (Appendix Fig S1A), and promoted a slight increase in the cell perimeter (Appendix Fig S1B). Moreover, Teneurin4^WT^ and Teneurin4^Mut^ protein overexpression resulted in an increase in the formation of filopodia (Fig 6A–D). The length of filopodia was only subtly increased when comparing Teneurin4^WT^ and Teneurin4^Mut^ to GFP control condition (Fig 6E, Appendix Fig S1C). Both proteins localized to the filopodia (Fig 6D) and were often specifically enriched at the filopodia tips (Fig 6B, arrowheads), suggesting a role in sensing the extracellular environment (Jacquemet *et al*, 2019). We found no correlation between the expression level of either protein and the number of filopodia per cell, excluding a possible artifact due to expression levels (Appendix Fig S1D). Altogether, these data demonstrate that Teneurin4^Mut^ retains its biological activity and that Teneurin4 may play a role in cell migration or adhesion by promoting the extension of filopodia.

### The compact Teneurin4 *cis* dimer supports trans‐interactions

**Figure 7:**
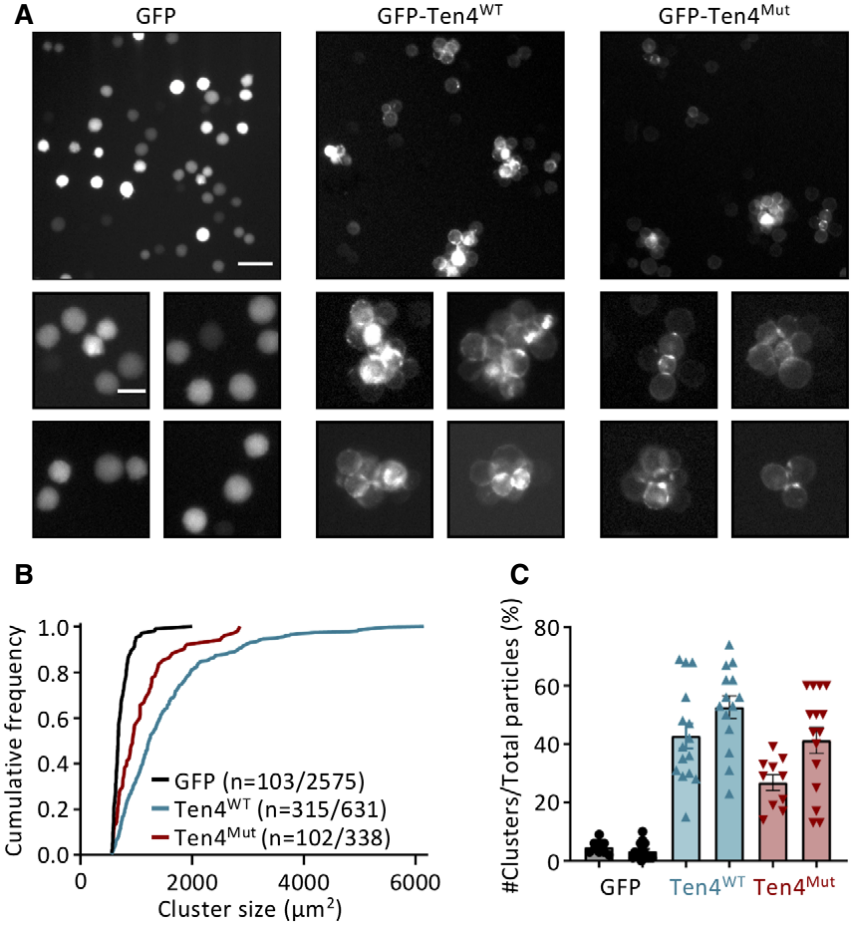
Overexpression of full‐length Teneurin4^WT^ and Teneurin4^Mut^ induces *trans*‐cellular interactions Representative images of nonadherent K562 cells electroporated with GFP (left), GFP‐Teneurin4^WT^ (GFP‐Ten4^WT^; middle) or GFP‐Teneurin4^Mut^ (GFP‐Ten4^Mut^; right). Zoomed images show the formation of cellular clusters in cells expressing GFP‐Ten4^WT^ and GFP‐Ten4^Mut^, but not in GFP‐expressing cells. Scale bar top panel, 50 µm. Scale bar bottom panel, 20 µm.Cumulative distributions of the cluster size in cells electroporated with GFP (black), GFP‐Teneurin4^WT^ (Ten4^WT^; blue) and GFP‐Teneurin4^Mut^ (Ten4^Mut^; red). Only particles with a cluster size above the average size of a large cell (~560 µm^2^) are included; *n* indicates the number of cell clusters/total particles.Fraction of cell clusters detected per image in GFP‐ (black), GFP‐Teneurin4^WT^ (blue)‐ and GFP‐Teneurin4^Mut^ (red)‐electroporated cells. Data information: Data are represented as mean ± SEM. Each dot represents one image. Data from 9 to 15 images per condition for each independent experiment.

It has been shown previously that Teneurin3 overexpression promotes cell clustering, indicating the formation of Teneurin *trans*‐dimers (Berns *et al*, 2018; Pederick *et al*, 2021). We set up a cell clustering assay to assess the role of Teneurin4 in *trans*‐interactions. Similar to Teneurin3, overexpression of Teneurin4^WT^ induced large K562 cell clusters, compared to only subtle clustering in the GFP‐only overexpression (Fig 7A and B). Besides, ~47% of the particles detected in the Teneurin4^WT^ overexpression are found in a cluster, while this is only 4% in the control GFP‐only overexpression (Fig 7C). Thus, Teneurin4^WT^ is capable of forming homomeric *trans*‐dimers in a cellular setting. Next, we tested whether Teneurin4^Mut^, in which the compact dimer interface (CYA‐CYA) is enforced, would abrogate *trans*‐dimerization. Our data show that Teneurin4^Mut^‐overexpressing K562 cells are also capable of clustering (Fig 7A–C). This indicates that the compact Teneurin4 conformation supports Teneurin4 *trans*‐interactions. Thus, the here proposed CYA‐CYA *cis*‐dimerization interface is itself not directly required for Teneurin4 *trans*‐interactions and the *trans*‐interactions may occur via a different interface.

### A model for trans‐synaptic interactions

**Figure 8:**
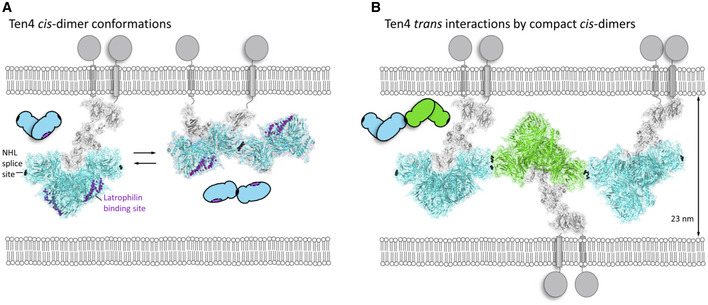
Model for Teneurin4 dimer conformations in *cis* and *trans* Two possible *cis* conformations of the Teneurin4 full‐ectodomain dimer as determined by cryo‐EM and SAXS modeling. The dimer subunits are covalently interacting through disulfide bonds in EGF2 and EGF5. The SAXS‐derived compact Teneurin4 dimer model harbors the CYA–CYA interface as we observed in cryo‐EM and SAXS in the presence of calcium (left). The SAXS‐derived elongated Teneurin4 dimer model harbors the NHL–NHL splice‐site‐dependent interface (Li *et al*, 2020), as based on SAXS data in the absence of calcium (right) (see Fig EV5F and G for SAXS modeling restraints and fit to the data). Both conformations may exchange and this is possibly calcium dependent. Cα atoms in the NHL splice site and Cα atoms of residues in the Latrophilin binding site are shown as black and purple spheres, respectively.Compact Teneurin4 *cis* dimers can interact in *trans* and may form an array. The dimension of the array in *trans* is ~23 nm. Homo‐*trans* interactions via the NHL splice site have previously been reported based on cellular assays for mouse Teneurin3 (Berns *et al*, 2018) and on crystal packing observed for the chick Teneurin2 superfold (Jackson *et al*, 2018). Cα atoms in the NHL splice site are indicated as black spheres. The *cis*‐*trans* model was generated by applying the crystal packing orientation of chick Teneurin2 (Jackson *et al*, 2018) onto the SAXS‐derived compact Teneurin4 ectodomain dimers. Data information: In panels A and B, the Ig and eight EGF domains are colored grey. Their structure and composition is based on homology modeling and SAXS data fitting and not represented by high‐resolution data. The superfold including the C‐rich domain is colored cyan and green and is represented by the cryo‐EM data presented in this study. The schematic drawings highlight the interactions between the Teneurin4 superfolds.

How is the dimer of Teneurin4 oriented toward the membrane? The two chains in the covalent Teneurin4 dimer will be on the same cell, that is the subunits interact in *cis* in the dimer through the intermolecular disulfide bonds in EGF2 and EGF5 (Feng *et al*, 2002). The most likely membrane orientation is with the EGFs facing the membrane (indicated in Fig 8A). In this orientation, the NHL domains are available for protein–protein interactions by facing outward in a ~90° angle with the membrane itself. The predicted dimensions of the modeled Teneurin4 ECD based on the SAXS data are 21 nm by 18 nm by 15 nm (see Figs 8A and EV5E). The synaptic gap width varies between 20 nm and 25 nm in intramembrane distance (Zuber *et al*, 2005; Harris & Weinberg, 2012; Tao *et al*, 2018). These dimensions would allow for Teneurin‐Latrophilin interactions in *trans* and the recently identified Latrophilin binding site on Teneurin (Del Toro *et al*, 2020; Li *et al*, 2020) is fully exposed in the Teneurin compact dimer structure (Fig 8A, left). A rearrangement from compact CYA‐CYA dimer to elongated NHL‐NHL dimer, as observed in the SAXS data of Teneurin4^WT^ in the absence of calcium, is compatible with the membrane attachment of full‐length Teneurin4 (Fig 8A, right). Notably, the SAXS (and cryo‐EM) derived compact *cis* dimer conformation, that interacts in *trans* in a homomeric manner (Fig 7), together with information from the crystallographic dimer as observed for chick Teneurin2 (Jackson *et al*, 2018) and the NHL splice‐site‐dependent Teneurin *trans*‐interaction (Berns *et al*, 2018) support a model for *cis*‐*trans* clustering of Teneurin4 (Fig 8B).

## Discussion

A comprehensive understanding of the role of Teneurins in the developing and adult CNS is steadily coming forth, revealing how Teneurins are required for specific targeted projections in multiple brain circuits. However, how the structural features of Teneurins enable initiation, specificity, or maintenance of these targeted projections is still unclear. Here, we show using cryo‐electron microscopy, X‐ray crystallography, small‐angle X‐ray scattering complemented with thermostability assays and *in vitro* cell culture assays that the full ectodomain of Teneurin4 can adopt a compact conformation that supports *trans*‐synaptic interactions.

The dimer interface in the compact conformation of Teneurin4 is established by interactions between the highly conserved ABD domain (see Fig EV3A and B) with the C‐rich region and the YD shell. As shown in Figs 3E and EV3, the domain‐specific architecture, including predicted glycans (Fig EV5C) is very well conserved between human Teneurin paralogs, indicating that the compact dimer configuration would be possible for the other Teneurin family members as well. Previous work, using rotary shadowing and negative stain EM, however has shown that covalent dimers of Teneurins are in an extended “cherry‐like” conformation (Oohashi *et al*, 1999; Feng *et al*, 2002; Li *et al*, 2020) in contrast to our compact Teneurin4 dimer conformation. We show by SAXS analysis that the presence of calcium is required for Teneurin4 to adopt a compact dimer conformation and we always supplemented our buffers with at least 1‐mM Ca^2+^ for structure determination. However, despite the presence of calcium, a large subset of Teneurin4^WT^ dimers seem to change to an extended conformation on the cryo‐EM grid (Fig EV1), presumably because of the air‐water interface (Glaeser & Han, 2017; Noble *et al*, 2018; D'Imprima *et al*, 2019). This effect is fully prevented by the Ser2585Cys mutation in Teneurin4^Mut^ in which the CYA–CYA interface is enforced. Possibly the lack of sufficient calcium and the sample preparation for EM also caused the Teneurin dimer to adopt an extended conformation in previous studies (Oohashi *et al*, 1999; Feng *et al*, 2002; Li *et al*, 2020). Nonetheless, we currently cannot exclude that the observed compact dimer is specific for Teneurin4, and does not occur for the other Teneurin paralogs.

Similar to Teneurin2 and Teneurin3, Teneurin4 may also be subject to alternative splicing. The Teneurin4 constructs used in this study contain the splice inserts between EGF7 and EGF8 and the NHL splice insert (also known as the A_1_B_1_ isoform). The location of the NHL slice insert (B_1_) is facing outward in the compact structure (Fig 8A, left). This splice site may be located in the elongated *cis* dimer interface (Fig 8A, right), as it explains the SAXS data of Teneurin4 in conditions that lack calcium (Fig EV5F and G) and it has previously been suggested to play a role in Teneurin2 *cis* dimerization (Li *et al*, 2020). The first splice insert A_1_, between EGF7 and EGF8, could potentially stabilize the compact dimer, but unfortunately the low resolution of the EGF domain in the Teneurin4 reconstruction limits further analysis.

What determines the balance between compact and extended covalent Teneurin dimer *in vivo*? The hydrophilic nature of the binding interface suggests that rearrangements from compact to extended are likely to occur. For instance, a change in configuration might be induced by binding a *cis‐* or *trans*‐interaction partner. The compact configuration is compatible with homomeric Teneurin4 *trans*‐interactions as the Teneurin4^Mut^ in which the CYA–CYA interface is enforced by the S2585C mutation induces cell clustering (Fig 7). In addition, the compact configuration seems to be compatible with *trans*‐synaptic Latrophilin binding as the Latrophilin binding site is exposed (Fig 8A), and also a ternary complex of Teneurin, Latrophilin, and FLRT may be possible if sufficient flexibility is present in the FLRT extracellular interdomain connections and in the N‐terminal region of the Teneurin ECD. However, quantitative analyses of *trans*‐membrane complex formation have so far been challenging, and cell biology binding studies from Li *et al*. already indicate that membrane‐bound constraints can alter Teneurin complex formation (Li *et al*, 2020). Future studies might reveal if the compactness of the covalent Teneurin dimer is indeed dependent on protein–protein interactions.

Filopodia, protrusion‐like extensions of the cell membrane, function as antennae to probe the environment, which is especially important during neurite outgrowth and dendritic spine formation (Lewis & Bridgman, 1992; Ozel *et al*, 2015, 2019). Teneurin2 and Teneurin4 have been described previously to induce filopodia formation in COS7 cells and Neuro‐2a neuroblastoma cells, respectively (Rubin *et al*, 2002; Suzuki *et al*, 2014). Teneurin4 specifically was observed at the growing tips of neurites (Suzuki *et al*, 2014). Neurite tips encompass an area with an approximate diameter of 5–10 µm (Suzuki *et al*, 2014). In contrast, filopodia are extended protrusions between 1 µm and 10 µm in length, with a diameter of 0.1–0.3 µm at the tip (Mattila & Lappalainen, 2008; Jacquemet *et al*, 2019). The filopodial tip localization observed in our data has not been described previously for Teneurin overexpression experiments. Generally speaking, the tip of a filopodium contains a specific subset of proteins, known as the tip complex, that is instrumental in physically linking the barbed ends of actin filaments and has a directive role in cell migration and adhesion (Svitkina *et al*, 2003). Such specific cellular localization of Teneurin proteins may hint toward a role in the initiation of synaptic contacts, which may contribute to the proper establishment of neuronal circuits in the mammalian brain.

Finally, risk variants and mutations in Teneurin4 have been associated with multiple pathological conditions, including Bipolar Disorder, Schizophrenia, and Essential Tremor (ET; Psychiatric, 2011; Muhleisen *et al*, 2014; Hor *et al*, 2015; Chao *et al*, 2016). The ET mutations can now be precisely mapped to the protein structure: the V1138 M mutation is located in strand 6 of the FN‐plug domain, the T1367N and A1442T mutations are located in respectively blade III and IV of the NHL domain. Residues V1138 and A1442 seem to have a structural role and may stabilize the FN‐plug and NHL domain, respectively, whereas residue T1367 is located at the surface of Teneurin4. A potential causative role of Teneurin4 in ET pathobiology is supported by the finding that mice lacking Teneurin4 suffer from myelination defects, resulting in an ET‐like phenotype (Suzuki *et al*, 2012; Hayashi *et al*, 2020). Future research is needed to pinpoint structure‐function relationships of the above‐mentioned mutants.

In conclusion, the compact covalent *cis* dimer of Teneurin4 could provide a scaffold for macromolecular complex assembly in the neuronal synapse in a Ca^2+^‐dependent manner. The compact arrangement is centered around the ABD domain, while presenting multiple other domains, including the NHL domain and the YD‐shell, for homomeric or heteromeric interactions. Future insights of whether other Teneurins are also capable of forming compact dimers, and what determines the balance between compact and extended dimer arrangement will aid our understanding of molecular guidance and recognition in neuronal circuit wiring.

## Materials and Methods

### Vectors and cloning

The plasmid encoding the full ECD of human Teneurin4 (residues 375–2,769) was created by subcloning a gBlocks Gene Fragment (residues 375–662, Integrated DNA Technologies) with the partial coding region of human Teneurin4 (residues 663–2,769, BC172403, Biocat) into the pUPE106.03 vector containing a N‐terminal cystatin secretion signal and N‐terminal His6 tag (U‐Protein Express) using BamH1, SfiI and NotI cloning sites. The plasmid encoding full‐length human Teneurin4 was created by subcloning a gBlocks Gene Fragment (residues 1–374, Integrated DNA Technologies) with the full ECD segment of Teneurin4 into the pUPE3620 vector containing a N‐terminal GFP tag using BamH1, Stu1, and NotI cloning sites. The plasmid encoding the C‐rich domain of human Teneurin4 (residues 834–871) was created from a template including restriction sites ordered from GeneArt (Thermo Fisher Scientific), which was subcloned into the pUPE106.03 vector containing a N‐terminal cystatin secretion signal and N‐terminal His6 tag (U‐Protein Express) using BamH1 and NotI cloning sites.

### Cell culture

Epstein–Barr virus nuclear antigen I‐expressing HEK293 cells (HEK‐E; U‐Protein Express) were cultured in FreeStyle293 expression medium with GlutaMAX (FreeStyle; Gibco) supplemented with 0.2% fetal bovine serum (FBS, Gibco) and 0.1% Geneticin (G418 Sulfate; Gibco). HEK293‐T cells were purchased from Leibniz Institute DSMZ‐German Collection of Microorganisms and Cell Cultures (ACC 635). HEK293‐T were cultured in DMEM/F12 with GlutaMAX (Gibco) supplemented with 10% FBS (Gibco) and 1% Penicillin/Streptomycin (Gibco). K562 cells were cultured in RPMI 1640 medium (Gibco), supplemented with 10% FBS (Gibco) and 1% Penicillin/Streptomycin (Gibco). All cell types were grown at 37°C and 5% CO_2_.

### Protein expression and purification

All proteins were expressed in a secreted form using transiently transfected HEK‐E cells. Prior to transfection, HEK‐E cells were seeded onto 1‐l Erlenmeyer cell culture flasks and grown in FreeStyle medium without supplements. 24 h after culture, cells were transfected with a total of 125 μg of DNA with 1 μg/μl polyethylenimine (PEI, Polysciences) per 250 ml of cell culture according to manufacturer’s protocol. HEK‐E cells were treated with 5.5% Primatone in FreeStyle medium 6–24 h posttransfection, and the medium was collected 6 days after transfection. Cell medium was centrifuged for 10 min at 300 g and filtered using a 0.45 µm filter. Medium was diafiltrated into 25‐mM HEPES (pH 7.8), 500‐mM NaCl and 2‐mM CaCl_2_. Proteins were purified by Ni‐NTA affinity chromatography using an elution buffer containing 25‐mM HEPES (pH 7.8), 500‐mM NaCl, 2‐mM CaCl_2,_ and 500‐mM Imidazole, followed by size‐exclusion chromatography using a Superose6 column (GE Healthcare) into a final buffer composition of 20‐mM HEPES (pH 7.8), 150‐mM NaCl, and 2‐mM CaCl_2_.

### Cryo‐electron microscopy

Purified human Teneurin4^WT^ and Teneurin4^Mut^ proteins were diluted to a final concentration of 75 μg/ml in 75‐mM NaCl, 1‐mM CaCl_2,_ and 10‐mM HEPES, pH 7.8. For human Teneurin4^WT^, Quantifoil R2/2 200 mesh grids were glow discharged for 35 s at 15 mA, after which 3.0 μl of diluted protein was applied. Grids were blotted for 3 s with a blot force of −3 using a Vitrobot Mark IV (Thermo Fisher Scientific). Data were acquired on a Krios microscope, equipped with a Falcon 3 camera (Thermo Fisher Scientific). Movies were collected at a nominal magnification of 75,000×, corresponding to a physical pixel size of 0.88 Å at the specimen level. The exposure time was 75.02 s resulting in a total dose of 50 e^−^/Å^2^ for the integrated image. A total of 3,970 micrographs were collected using EPU software (Thermo Fisher Scientific), with a defocus range of −1.1 to −2.8 μm. For human Teneurin4^Mut^, Quantifoil R2/2 200 mesh grids were glow discharged for 45 s at 45mA, after which 3.5‐μl diluted protein was applied. Grids were blotted for 2 s with a blot force of −3 using a Vitrobot Mark IV (Thermo Fisher Scientific). Movies were acquired on a Krios microscope, equipped with a K2 camera and GIF quantum energy filter (Gatan) using a 20 eV slit width and operated in counting mode. Data were collected at a nominal magnification of 165,000×, corresponding to a physical pixel size of 0.84 Å at the specimen level. The exposure time was 3.4 s resulting in a total dose of 50.6 e^−^/Å^2^ for the integrated image. A total of 2,648 micrographs were collected using EPU software (Thermo Fisher Scientific), with defocus ranging from −0.75 to −1.75 μm. For both datasets, all micrographs were corrected for beam‐induced motion and drift using MotionCor2 (Zheng *et al*, 2017). After CTF modeling with Gctf (Zhang, 2016), particles were picked using the EMAN2 neural network particle picking (Bell *et al*, 2018). For the Teneurin4^WT^ dataset, extracted particles with box size of 275 Å (312 pixels) were subjected to 2D classification in Relion3.1 (Zivanov *et al*, 2020) using a spherical mask, with a diameter of 260 Å. For the Teneurin4^Mut^ dataset, particles were extracted with a box size of 302 Å (360 pixels), no 2D classification selection step was performed. For the Teneurin4^WT^ dataset an ab‐initio 3D reference map was generated in Relion3.1. For both datasets, 3D map reconstructions were performed according to the regular workflows of Relion3.1. Symmetry expansion, from C2 to C1, was applied to the refined Teneurin4^Mut^ dataset and followed by focused refinement on a single Teneurin4 subunit. To improve the resolution of the C‐rich domain, first a 3D classification without alignment was done on the symmetry‐expanded reconstruction based on C‐rich – TTR (~20 kDa volume) and using a regularization parameter of 40 in Relion3.1. Out of ten classes, four were refined that had clear density for the C‐rich domain, and these maps revealed four different positions of the C‐rich–TTR combination with respect to the Teneurin4 superfold, indicating that the C‐rich–TTR combination is slightly mobile. The superposition matrixes, determined in Chimera, for the C‐rich domain in the four independent maps were applied to their half‐maps and the transformed half maps were resampled on their original grid in Chimera. The four superposed half1 maps and the four superposed half2 maps were summed in Chimera, providing two independent summed half‐maps that were used for postprocessing in Relion3.1. The resulting half‐maps of all the final reconstructions were used to calculate the Fourier Shell Correlation (FSC) by generating soft‐edge extended masks. Local resolutions were determined by the local resolution algorithm in Relion3.1.

### Model building and refinement

A model of Teneurin4 based on the crystal structure of Teneurin2 (Jackson *et al*, 2018) was generated in SWISS‐MODEL (Waterhouse *et al*, 2018) and partially refined against the Teneurin4^WT^ map by iterative cycles of manual model building in Coot (Emsley & Cowtan, 2004) and real space refinement in phenix.real_space_refine of the Phenix package (Adams *et al*, 2010). The model was further optimized against the symmetry‐expanded Teneurin4^Mut^ dataset using Coot and Phenix. At the final stages, this optimized model was refined in the dimer maps of Teneurin4^Mut^ and Teneurin4^WT^ using C2 symmetry constraints in Phenix. The crystal structure model of the C‐rich domain that we determined to 1.5 Å resolution was used to model the C‐rich domain in all the EM maps. The stereochemistry of the models was checked with MolProbity (Williams *et al*, 2018), the interfaces were analyzed with the Protein Interfaces, Surfaces and Assemblies (PISA) web server (Krissinel & Henrick, 2007), r.m.s.d. of atomic positions were calculated using SUPERPOSE in CCP4 (Krissinel & Henrick, 2004) and alignments were scored in Clustal Omega (Sievers *et al*, 2011). Evolutionary conservation scores were calculated with ConSurf, using the UniRef90 database to identify homologous sequences (Ashkenazy *et al*, 2016). The structural figures of maps and models were generated with Chimera (Pettersen *et al*, 2004) and PyMOL (Schrodinger Molecular Graphics System, DeLano Scientific, San Carlos, CA).

### Crystallization and data collection

Crystallization trials were set up using purified C‐rich domain, at a concentration of 9 mg/ml. This yielded crystals in a condition of 0.085 M HEPES pH 7.5, 1.7 v/v % PEG‐400, 1.7 M (NH_4_)_2_SO_4_ and 15 w/v % glycerol, at a ratio of two parts protein and one part reservoir solution, at 20°C. Crystals were frozen in a mixture of 4:1 reservoir solution and glycerol. Datasets were collected at 100 K at the European Synchrotron Radiation Facility (ESRF) beamline ID23‐1. A native dataset was collected at a wavelength of 0.7749 Å and an anomalous dataset was collected at a wavelength of 1.771 Å.

### Structure determination and refinement

Data were integrated using the EDNA_proc and XIA2_DIALS pipelines for the native and anomalous datasets, respectively. For both datasets, data were merged and scaled in AIMLESS. The dataset statistics are reported in Table 3. The substructure of the anomalous scatterers in the anomalous dataset was determined and the dataset was phased in the CRANK2 pipeline (Skubak & Pannu, 2013). An initial model was built automatically from the phased anomalous dataset using Buccaneer and refined using REFMAC5 (Kovalevskiy *et al*, 2018) in the CRANK2 pipeline. This model was directly used for molecular replacement in the native dataset using PHASER (McCoy *et al*, 2007) The structure was refined by iterative rounds of manual model building in COOT (Emsley & Cowtan, 2004) and refinement in REFMAC5 (Kovalevskiy *et al*, 2018) and ran through the PDB_REDO server (Joosten *et al*, 2014) in between refinement rounds once. The final model was assessed using MolProbity (Williams *et al*, 2018). All programs were used as implemented in CCP4i2 version 1.0.2 (Winn *et al*, 2011).

### Thermal shift assay

Thermal shift assay (TSA) assays were performed using purified human Teneurin4^WT^ and Teneurin4^Mut^ at concentration of 0.5 mg/ml. Prior to the experiments, the samples were buffer exchanged by SEC into SEC buffer without additional calcium (150‐mM NaCl, 20‐mM HEPES, pH 7.8) denoted “low calcium” condition (+/− Ca^2+^) and supplemented with 2‐mM CaCl_2_ (physiological calcium; + Ca^2+^) or 2 mM EDTA (no calcium; − Ca^2+^) as indicated. SYPRO Orange Dye (Invitrogen) was diluted to a concentration of 5X concentrated solution and filtered using a 0.2 μm membrane. Final concentrations were 60 μg/ml protein, 0.6× dye and final buffer concentrations 132‐mM NaCl, 17.6‐mM HEPES, pH 7.8, supplemented with 1.76‐mM CaCl_2_ or EDTA. A temperature ramp from 5°C to 95°C was set up at a speed of 0.02°C/s on a QuantStudio 3 Real‐Time PCR system (Thermo Fisher Scientific). All measurements were performed in triplicates.

### Small‐angle X‐ray scattering

SAXS was performed at the Diamond Light Source B21 beamline, at 13.1 keV operating energy equipped with an Eiger 4 M detector at detector distance of 3.5 m. Thirty‐five microliters of the samples at 1 mg/ml, prepared as described for the TSA assay, were injected and data were collected at 20°C over 25 frames, one frame per second, with a scattering vector range of 0.0026–0.34 Å^−1^. Radiation damage was monitored and data frames were selected manually in the PRIMUS GUI (Konarev *et al*, 2003), which was also used for frame averaging, buffer subtraction, Guinier modeling, and determining the pair‐distance distribution P(r). Kratky analyses were performed according to Durand *et al* (2010). Partial structures for the EGF domains were modeled using Phyre2 (template: 2E26; confidence > 98.5; Kelley *et al*, 2015), and the predicted Ig‐fold was modeled using trRosetta (Yang *et al*, 2020) and confirmed by AlphaFold, with r.m.s.d. 1.9 Å over 99 Cα atoms (Jumper *et al*, 2021). Complex glycans were added to predicted N‐glycosylated residues using GLYCOSYLATION (Petoukhov & Svergun, 2005). Together with the superfold model determined by EM, these models were used as input for rigid‐body modeling of two chains, that is a total of 20 rigid bodies with short flexible linkers, using CORAL (Petoukhov *et al*, 2012). For modeling purposes, scattering data were truncated to exclude low‐resolution (based on Guinier analysis cut‐off) and high‐resolution data points with low signal‐to‐noise ratios. The dimerization interfaces observed in the EM model or crystal packing of chick Teneurin2 were constrained using distance constrains; the putative cysteine bonds between EGF2 and EGF2 or EGF5 of the opposing chain were also represented using distance constrains. All χ^2^ fits were calculated using CRYSOL 2.8.4 via PRIMUS (Svergun *et al*, 1995). From the best fitting model, the dimerizing EGFs (2–5) were taken as a single rigid body and, supplemented with the other domains to model two chains, that is, a total of 13 rigid bodies with flexible linkers, used as inputs for generation of a random pool of proteins by EOM 2.0 (Bernado *et al*, 2007; Tria *et al*, 2015). Ensembles consisting of these structures fitting to the SAXS data were then determined by GAJOE (Bernado *et al*, 2007; Tria *et al*, 2015).

### Heterologous cell transfection and stainings

Prior to transfection, adherent HEK293‐T cells were split and seeded onto 18 mm glass coverslips coated with 0.1% gelatin. HEK293‐T cells were transfected with a total of 2 μg of DNA per condition using 1 μg/μl PEI according to manufacturer’s protocol and fixed 24 h after transfection with 4% (w/v) paraformaldehyde (PFA; BosterBio) for 10 min. After fixation, cells were thoroughly washed three times for 5 min with phosphate buffer saline (1× PBS) and permeabilized with 0.5% Triton X‐100 in PBS for 10 min. Cells were then blocked with 5% normal goat serum (Life Technologies) and 0.05% Tween20 in PBS for 30 min. Cells were incubated with Alexa 568 Phalloidin (A12380, Invitrogen) in blocking solution for 1 h at room temperature. After washing three times in 1× PBS and a final wash in MilliQ, coverslips were mounted on slides in Vectashield mounting medium with Dapi (Vector Laboratories).

### Confocal imaging and analysis

High‐resolution confocal laser scanning microscopy was performed on a Nikon Eclipse Ti inverted system with a Plan‐Apochromat 100×/1.4 NA SR Apo Tirf oil‐immersion objective, using 405, 488, and 561 nm laser lines. Each image was a z‐series of 12–22 images (1 µm z‐step size). The imaging area was 125.58 µm × 125.58 µm (1,024 × 1,024 pixels). Settings were kept the same in each experiment.

Analysis was performed using Fiji (Schindelin *et al*, 2012). A cell mask was manually traced in maximum intensity projections and used to calculate the cell perimeter. Individual filopodia were manually traced from the filopodium tip until the cell membrane by inspecting all z‐sections. Only filopodia longer than 1 µm were considered for analysis. Mean GFP intensity was quantified inside the cell mask in average intensity projections and background values were subtracted. Data are represented as mean ± SEM. Results of two independent experiments are shown as individual bars. The total number of cells analyzed per condition were 32–33.

### Cell‐surface live‐staining, fixation, and imaging

For the cell surface live‐staining, we used MemBrite^®^ Fix 568/580 (Biotium). HEK293‐T cells were cultured onto 0.1% gelatin‐coated coverslips and transfected 24 h prior to the cell surface live‐staining as described before. The medium of the cells was removed and cells were washed with prewarmed 1x PBS. Prestaining solution was diluted in prewarmed 1x PBS, and added to the cells and incubated for 5 min at 37°C and 5% CO_2_. After, the prestaining solution was removed and cells were stained with MemBrite Fix dye diluted in 1× PBS for 5 min at 37°C and 5% CO_2_. Cells were then washed two times with 1× PBS and fixed with 4% PFA for 10 min at room temperature. After fixation, cells were washed three times for 5 min with 1× PBS and coverslips were mounted on slides with Vectashield mounting medium (Vector Laboratories). Confocal imaging was performed as described previously, with the difference of 0.5 µm z‐step size (each stack was a z‐series of 31–47 images).

### Cell clustering assay, imaging, and analysis

K562 cells were counted to obtain desired cell number and centrifuged for 5 min at 300 g. The cells were washed in 1× PBS (Gibco), then centrifuged again for 5 min at 300 *g* and resuspended in Opti‐MEM (Gibco). Per condition, 2 × 10^6^ cells were incubated with a total amount of 15 µg of DNA for 15 min at room temperature. The ratio of Teneurin4^WT^/Teneurin4^Mut^ or GFP (control) construct to empty vector was 1:10. After the incubation, the cell and DNA mixture was transferred into a 0.2‐cm cuvette (Sigma‐Aldrich) and electroporated using a Gene Pulser Xcell Electroporation System (Bio‐Rad Laboratories). The following parameters were used: 5 square‐wave pulses, each of 25 ms, 110V, 0.1 s interval. Cells were then added to 5 ml of prewarmed RPMI‐1640 with 10% FBS in a 6‐well plate and incubated for approximately 20 h at 37°C and 5% CO_2_. Cells were imaged on an EVOS M5000 microscope with a 10× objective, using the EVOS LED GFP cube (ThermoFisher Scientific). For the analysis, the background was removed from the GFP channel and images were then thresholded using Fiji (Schindelin *et al*, 2012). Analyze Particles (Fiji) was used to detect and measure cell size, and only particles above 150 µm^2^ were counted to detect all cells. To filter out the majority of single cells, the average size of large single cells was determined in the GFP (control) condition. Any particle above this average was considered a cell cluster. The fraction of cell clusters of two independent experiments is shown as individual bars and the total number of images analyzed per condition varied between 24 and 29. Data are represented as mean ± SEM.

## Author contributions

**Bert J C Janssen:** Conceptualization; Supervision; Writing—original draft; Writing—review and editing. **Dimphna H Meijer:** Conceptualization; Supervision; Investigation; Methodology; Writing—original draft; Writing—review and editing. **Cátia P Frias:** Investigation; Methodology; Writing—original draft; Writing—review and editing. **J Wouter Beugelink:** Investigation; Methodology; Writing—original draft; Writing—review and editing. **Yanthi N Deurloo:** Methodology.

In addition to the CRediT author contributions listed above, the contributions in detail are:

DHM, CPF, JWB, and YND designed and performed the experiments. DHM and BJCJ supervised the research. All authors were involved with data analysis. DHM and BJCJ wrote the manuscript in consultation with CPF and JWB.

## Supporting information



Appendix

Expanded View Figures PDF

## Data Availability

Cryo‐EM data have been deposited to the EMDB and PDB databases, with the following identifiers: EMD‐12122 (http://www.ebi.ac.uk/pdbe/entry/EMD‐12122), EMD‐12123 (http://www.ebi.ac.uk/pdbe/entry/EMD‐12123), EMD‐12124 (http://www.ebi.ac.uk/pdbe/entry/EMD‐12124), EMD‐12125 (http://www.ebi.ac.uk/pdbe/entry/EMD‐12125), EMD‐12126 (http://www.ebi.ac.uk/pdbe/entry/EMD‐12126) and EMD‐12127 (http://www.ebi.ac.uk/pdbe/entry/EMD‐12127); 7BAM (http://identifiers.org/pdb/7BAM), 7BAN (http://identifiers.org/pdb/7BAN), and 7BAO (http://identifiers.org/pdb/7BAO). X‐ray data have been deposited to the PDB database, with identifier 7PLP (http://identifiers.org/pdb/7PLP). SAXS data have been deposited to the SASDB databases and assigned the identifiers SASDKS4 to SASDKX4 (http://www.sasbdb.org/project/1220/). See Tables 1, 3, and 4 for specifications.
