# Supplementary material for: Teneurin4 dimer structures reveal a calcium‐stabilized compact conformation supporting homomeric trans‐interactions
Source: EMBO J. 2022 Jan 31;41(9):e107505. doi: 10.15252/embj.2020107505 (PMC9058538; doi:10.15252/embj.2020107505)
Supplement: Supplementary file 1 — Appendix [file EMBJ-41-e107505-s002.pdf]

# Appendix Table of Contents

1. Appendix Figure S1 **Related to Figure 6**. **Teneurin4$^{WT}$ and Teneurin4$^{Mut}$ localize to the cell membrane and overexpression slightly increases cell perimeter**

Meijer et al., Appendix Figure S1

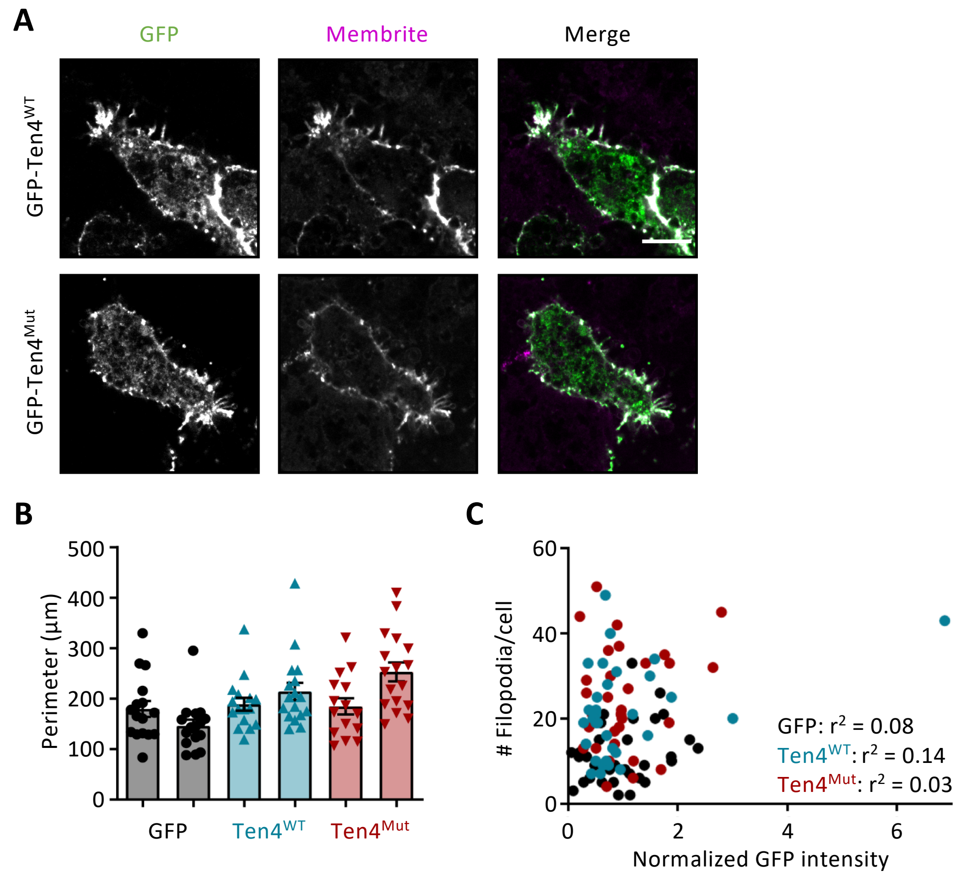


Appendix Figure S1 **Related to Figure 6**. **Teneurin4$^{WT}$ and Teneurin4$^{Mut}$ localize to the cell membrane and overexpression slightly increases cell perimeter**

**(A)** Representative images of HEK293-T cells transfected with GFP-Teneurin4$^{WT}$ (GFP-Ten4$^{WT}$; top) or GFP-Teneurin4$^{Mut}$ (GFP-Ten4$^{Mut}$; bottom) for 24h and stained with Membrite Fix 568/580 (magenta). Images are a single z plane. Scale bar, 10 µm.

**(B)** Perimeter of cells transfected with GFP, GFP-Teneurin4$^{WT}$ (Ten4$^{WT}$) and GFP-Teneurin4$^{Mut}$ (GFP-Ten4$^{Mut}$).

Data information: Each bar represents an individual experiment and each symbol represents an individual cell. Data are represented as mean ± SEM.

**(C)** Correlation between the normalized GFP intensity and the number of filopodia per cell in cells transfected with GFP (black; $r^2$ = 0.08), GFP-Teneurin4$^{WT}$ (blue; $r^2$ = 0.14) and GFP-Teneurin4$^{Mut}$ (red; $r^2$ = 0.03).

Data information: In (B) and (C), data from 32-33 cells per condition from two independent experiments.
