# Supplementary material for: Teneurin4 dimer structures reveal a calcium‐stabilized compact conformation supporting homomeric trans‐interactions
Source: EMBO J. 2022 Jan 31;41(9):e107505. doi: 10.15252/embj.2020107505 (PMC9058538; doi:10.15252/embj.2020107505)
Supplement: Supplementary file 2 — Expanded View Figures PDF [file EMBJ-41-e107505-s001.pdf]

Expanded View Figures

Figure EV1. Related to Fig 1. Image reconstruction of Teneurin4^{WT}.

A Size-exclusion chromatography traces of purified Teneurin4^{WT} dimer (blue) and Teneurin4^{Mut} dimer (red). The difference in peak retention volume may indicate a size difference of the two Teneurin4 samples, with a smaller (or more compact) size for the Teneurin4^{Mut} dimer. Inset shows Coomassie-blue-stained SDS-Page gel in presence and absence of β-mercaptoethanol (BME) and confirms both samples are covalent dimers.
B Representative electron micrograph of Teneurin4^{WT}. Scale bar, 40 nm.
C Selected 2D classes of Teneurin4^{WT}. Scale bar, 10 nm.
D Workflow for Teneurin4 reconstruction. Note that a small subset of particles represents compact dimers, and a large subset represents extended dimers that have different conformations resulting in the reconstruction of a single subunit only.
E C1 symmetry reconstruction of Teneurin4^{WT} compact dimer colored by local resolution as in (G).
F C2 symmetry reconstruction of Teneurin4^{WT} compact dimer colored by local resolution as in (G).
G Reconstruction of Teneurin4^{WT} core colored according to local resolution.
H Same as (E), but rotated by 180° over the x-axis.
I Structure of Teneurin4^{WT} in cartoon representation with EGF repeat 6–8 depicted as density maps.
J Fourier Shell Correlation of structures shown in E–G. Dotted line represents FSC = 0.143.

© 2022 The Authors

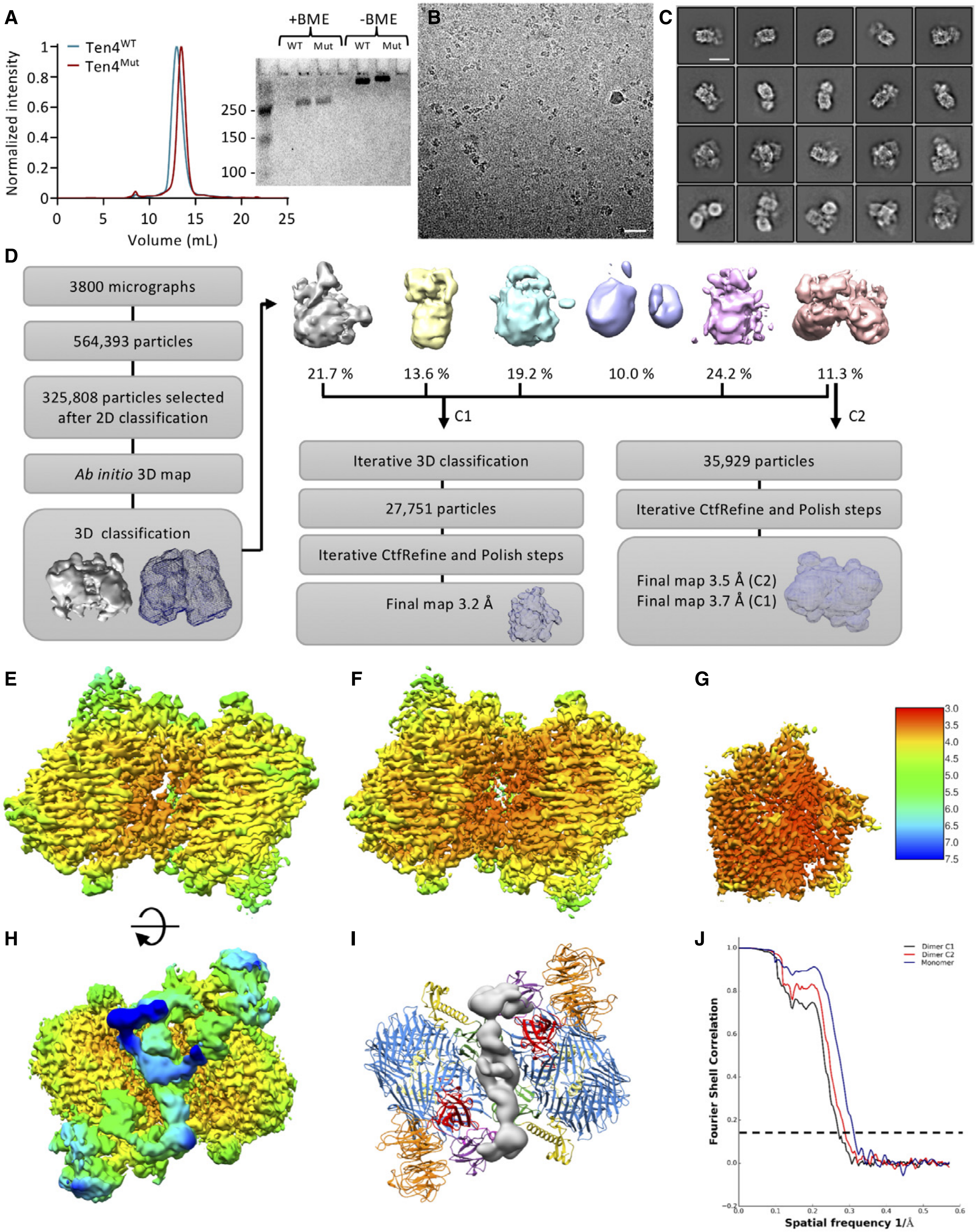

Figure EV1.

© 2022 The Authors

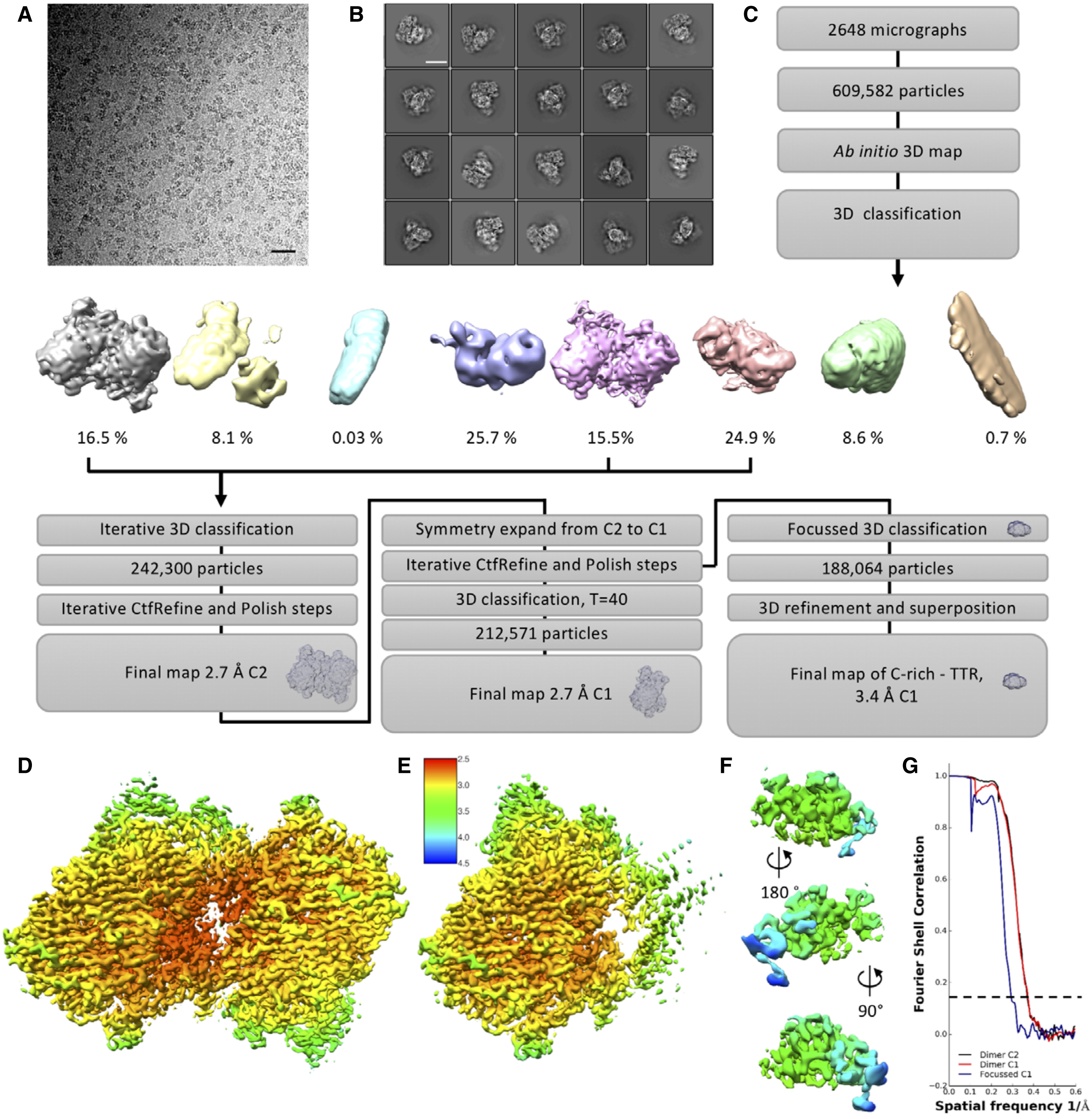

Figure EV2. Related to Fig 1. Image reconstruction of Teneurin4^{Mut}.

A Representative electron micrograph of Teneurin4^{Mut}. Scale bar, 40 nm.
B Selected 2D classes of Teneurin4^{Mut}. Scale bar, 10 nm.
C Workflow for Teneurin4^{Mut} reconstruction.
D C2 symmetry reconstruction of Teneurin4^{Mut} colored by local resolution. Coloring as in (E).
E Symmetry expanded half-dimer reconstruction of Teneurin4^{Mut} colored by local resolution. The map quality of the C-rich region, TTR, FN-plug and NHL domains is improved in this reconstruction.
F Three different side views of a focused reconstruction of Teneurin4^{Mut} C-rich–TTR. Top panel same orientation as D and E.
G Fourier Shell Correlation of structures shown in D–F. Dotted line represents FSC = 0.143.

© 2022 The Authors

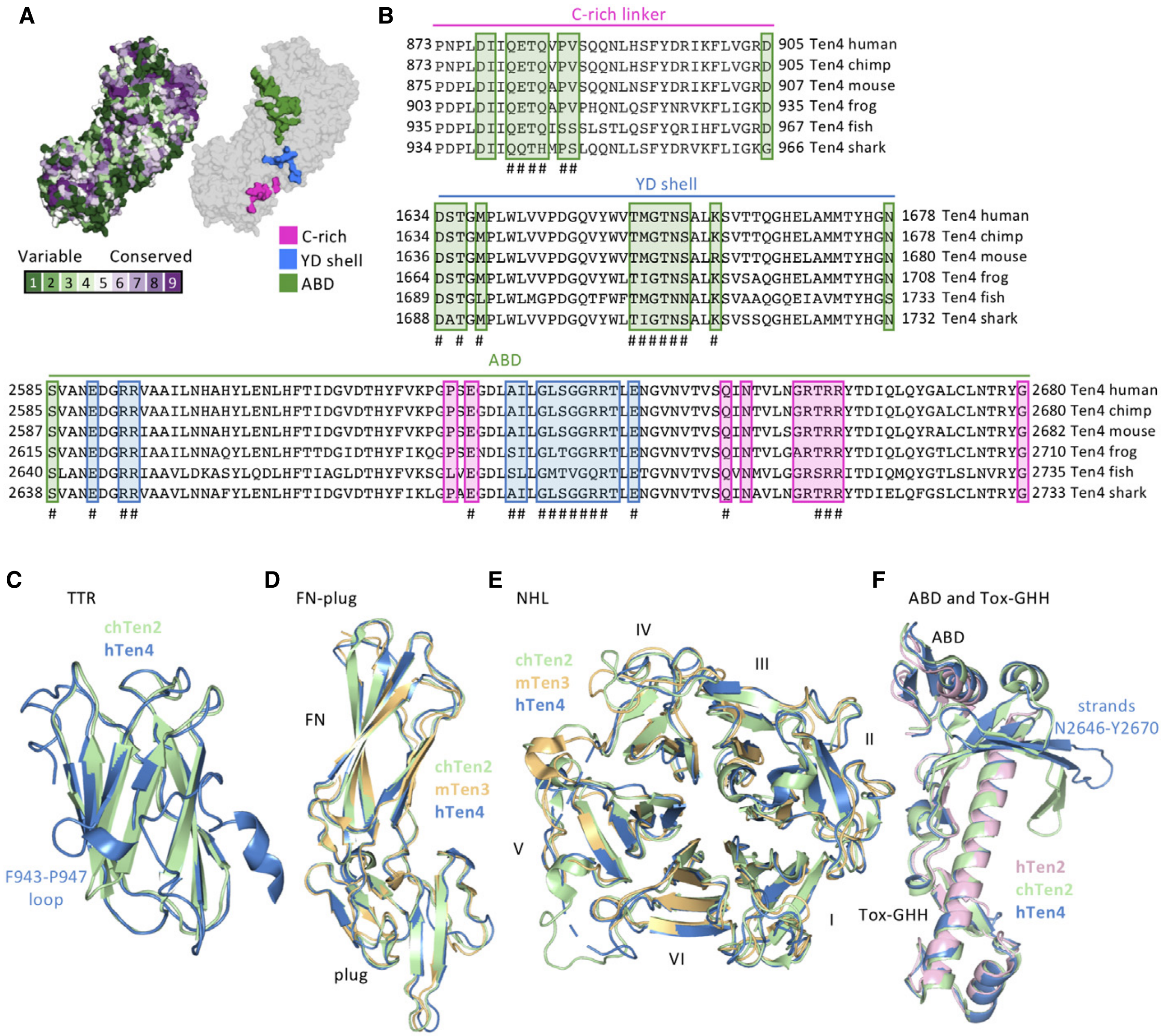

Figure EV3. Related to Figs 2 and 3. Domain-specific comparisons of published Teneurin structures.

- A Surface representation of evolutionary conserved score (left panel) and dimer interface residues (right panel), colored as in Fig 1A.
- B Amino acid alignment of interface residues in vertebrates. Coloring as in Fig 2D.
- C Overlay of cartoon representations of the TTR domain of chick Teneurin2 (6FB3; green) and human Teneurin4 (7BAM; blue), r.m.s.d. 1.0 Å for 80 Cα atoms.
- D Overlay of cartoon representations of the FN-plug domain of chick Teneurin2 (6FB3; green), mouse Teneurin3 (6FAY; orange) and human Teneurin4 (7BAM; blue), r.m.s.d. 0.8 Å over 185 Cα atoms for chick Teneurin2 and human Teneurin4, and 1.4 Å over 185 Cα atoms for mouse Teneurin3 and human Teneurin 4.
- E Overlay of cartoon representations of the NHL domain of chick Teneurin2 (6FB3; green), mouse Teneurin3 (6FAY; orange) and human Teneurin4 (7BAM; blue). Highest r.m.s.d. 1.2 Å over 326 Cα atoms for mouse Teneurin3 and human Teneurin 4.
- F Overlay of cartoon representations of the ABD and Tox-GHH domains of human Teneurin2 (6CMX; pink), chick Teneurin2 (6FB3; green) and human Teneurin4 (blue). R.m.s.d. of both domains equals 1.0 Å over 135 Cα atoms for human Teneurin2 and human Teneurin4, and 0.8 Å over 189 Cα atoms for chick Teneurin2 and human Teneurin4.

 © 2022 The Authors

Figure EV4. Related to Fig 4. C-rich domain calcium coordination and homology.

A,B Octahedral coordination of calcium ions (A) I, II, and (B) III by (mostly) negatively charged residues or backbone carbonyl oxygens of labeled residues. Calcium-coordinating bond distances are indicated in Å and consistent with expected calcium coordination.

C Coordination of two calcium ions (I and II) in the C-rich domain of Teneurin4 (pink) matches with the coordination of two calcium ions in thrombospondin type 3 (T3) repeats (green, Thrombospondin 1 - TSP-1; PDB 1UX6; Kvansakul *et al*, 2004).

D Coordination of the third calcium ion (labeled III) in the Teneurin4^{WT} C-rich domain matches with the coordination of the calcium ion in the low-density lipoprotein receptor (LDLR) class A (LA1) modules (orange, Apolipoprotein E receptor 2-ApoER2; PDB 3A7Q; Yasui *et al*, 2010).

E Part of the C-terminal side of the C-rich domain (residues 855-864) has structural similarity to conotoxins (light brown, A-family conotoxin; PDB 7EDK). The calcium ions (I-III) in the Teneurin4^{WT} C-rich domain are shown for reference.

F Cryo-EM density map (see also EV2F) of the C-rich domain and TTR region. Orientation of the C-rich domain is similar as in Fig 4E.

G Intramolecular C-rich domain–TTR interaction. Residues at the interface are labeled.

H Intramolecular C-rich linker–TTR interaction. Residues at the interface are labeled.

I Disulfide bond forming cysteine residues (bold and black) and calcium coordinating acidic residues (bold and red) are fully conserved among Teneurins in the animal kingdom ranging from choanoflagellate and worm through to mammals.

J None of the Teneurin4 EGFs contain the canonical calcium-binding EGF signature sequence. Sequence alignment of human Teneurin4 EGF domains, compared to a non-calcium-binding EGF domain from mouse Reelin (UNIPROT: Q60841) and a calcium-binding EGF domain from human Factor IX (UNIPROT: P00740), as well as the regular and calcium-binding EGF signature as defined by PROSITE. Residues directly involved in calcium-binding are indicated in blue, while cysteines are indicated in yellow. Residues that agree with the signatures are indicated in bold. Lack of the calcium binding signature in all of the human Teneurin4 EGFs may indicate that the Teneurin4 EGFs are not involved in calcium binding although a role for the EGFs in calcium binding cannot be fully excluded.

© 2022 The Authors

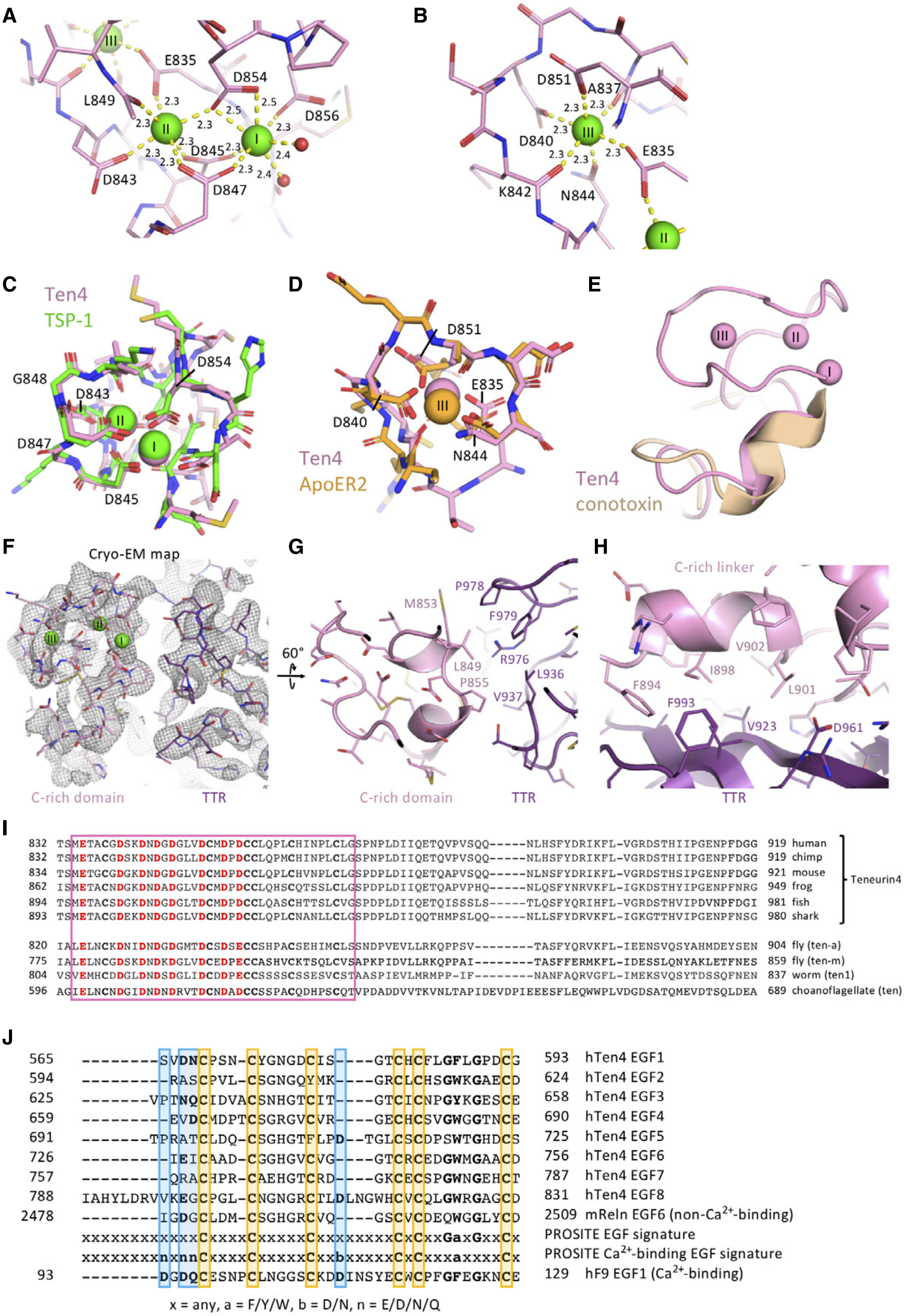

Figure EV4.

 © 2022 The Authors

Figure EV5. Related to Fig 5. Teneurin4^{WT} and Teneurin4^{Mut} are compact *cis* dimers in the presence of physiological calcium.

A SAXS Log I versus q plots for Teneurin4^{WT} (left) and Teneurin4^{Mut} (right) in physiological calcium concentrations (+ Ca^{2+}; black), low calcium concentrations (+/− Ca^{2+}; blue and red, respectively) and no calcium (− Ca^{2+}; grey).

B SAXS-based Guinier plots for Teneurin4 proteins colored as in A.

C Table indicating predicted sugars positions according to the NetNGlyc server for human Teneurin proteins (Uniprot codes Teneurin1, Q9UKZ4; Teneurin2, Q9NT68; Teneurin3, Q9P273; Teneurin4, Q6N022).

D Table indicating the χ^2 values of the SAXS data comparison of Teneurin4^{WT} and Teneurin4^{Mut} ECD in different calcium conditions.

E Cartoon representation of the best-fitting SAXS-based rigid-body model, same as in Fig 5C, inset. The two different superfolds in the covalent dimer are colored green and cyan, respectively. The Ig-like and EFG domains, placed by SAXS-based rigid body modeling, are colored light and dark grey to indicated the two chains in the dimer. Modeled glycans are in stick representation. Modeling parameters for this model: Ten4^{WT}, + Ca^{2+}, 2-5/5-2 EGFs, CYA-CYA interface.

F–G Models of the full Teneurin4 ectodomain were generated using CORAL (Petoukhov *et al*, 2012). The CORAL-based rigid body models inform on how well the models fit (or do not fit) the SAXS data. The EGF and Ig domain stalk, colored grey in panel E and Fig 8, represents the best fitting conformation but other conformations of the EGF-Ig stalk also fit the data. The scattering data of the Teneurin4^{WT} protein in either no calcium or physiological calcium concentration were used for modelling, also indicated in the comparison table in bold. To model *cis* dimers, interactions were enforced between the EGFs and superfolds of the intermolecular chains using distance restraints, indicated in cartoons in G. For the EGFs, inter-dimer covalent disulfide bonds were restrained either from EGF2 to EGF2 and EGF5 to EGF5 from one chain to the other as in a "parallel" fashion, or from EGF2 to EGF5 and EGF5 to EGF2, as in an "anti-parallel" fashion. Superfolds of opposite chains were restrained to form the C-rich/YD shell/ABD interface (CYA; this paper), or a previously described interface (NHL-NHL interface), involving a splice site in the NHL domain, resulting in more elongated particles (Jackson *et al*, 2018; Li *et al*, 2020). In addition, the effect was assessed of fixing the position of EGF7 and EGF8 with regard to the superfold, based on the low-thresholded C1 EM map (indicated by a star). χ^2 comparison of models to data, as calculated by CRYSOL (Svergun *et al*, 1995), are indicated at the right side of the table. There was no substantial difference in comparing model to data for the two EGF *cis*-dimerization modes. In addition, models obtained by fixing the positions of EGF-7 and 8 with regard to the Teneurin4 superfold, compared to not fixing those positions, explain the scattering data at physiological calcium concentrations equally well, again indicating that the SAXS and cryo-EM data in the presence of calcium are very similar. *Trans* models, in which two Teneurin4 *cis* dimers interact, do not fit to the SAXS data as the rigid body modelling leads to severe steric classes.

A

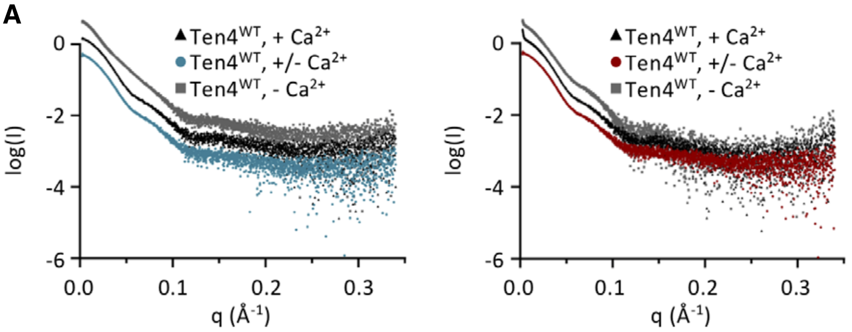

B

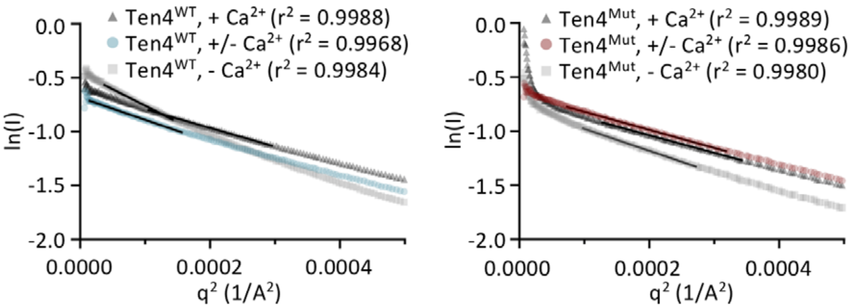

C

hTen1	hTen2	hTen3	hTen4
-	-	345	-
-	443	380	-
433	482	419	467
-	-	670	-
-	925	860	-
905	948	883	940
1084	-	-	-
-	1267	1202	1259
1550	-	1527	-
1567	1616	1544	1609
1663	1712	1640	1705
1699	1749	1677	1741
-	1773	-	-
1757	1807	1735	1799
1781	-	-	-
1842	1892	1820	1884
-	1993	1921	1985
2145	2197	2124	2188
2285	2337	2264	2328
2602	2648	2576	2646

D

χ^2 values	WT + Ca^2	WT +/- Ca^{2+}	WT - Ca^{2+}	Mut + Ca^2	Mut +/- Ca^{2+}	Mut - Ca^{2+}
WT, + Ca^2	-	-	-	-	-	-
WT, +/- Ca^{2+}	0.90	-	-	-	-	-
WT, - Ca^{2+}	90.8	86.7	-	-	-	-
Mut, + Ca^2	1.99	2.80	109.3	-	-	-
Mut, +/- Ca^{2+}	1.42	2.19	104.2	1.13	-	-
Mut, - Ca^{2+}	5.97	4.61	39.8	9.10	9.50	-

E

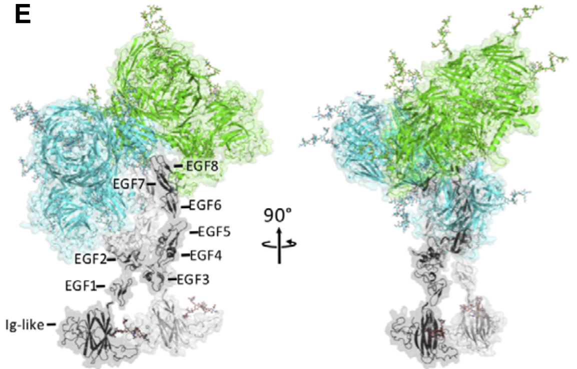

F

SAXS modelling restraints			χ^2 comparisons of models to SAXS data				
SAXS Tenm4^{WT} [Ca^{2+}]	Dimer interactions: EGF	Dimer interactions: Superfold	Tenm4^{WT} - Ca2+	Tenm4^{WT} + Ca2	Tenm4^{WT} +/- Ca2+	Tenm4^{MUT} + Ca2	Tenm4^{MUT} +/- Ca2+
-	2-2 / 5-5	NHL – NHL	**1.70**	-	-	-	-
-	2-5 / 5-2	NHL – NHL	**2.51**	-	-	-	-
-	2-2 / 5-5	CYA – CYA	**28.62**	-	-	-	-
-	2-5 / 5-2	CYA – CYA	**28.94**	-	-	-	-
+	2-2 / 5-5	NHL – NHL	-	**14.78**	10.45	22.73	23.51
+	2-5 / 5-2	NHL – NHL	-	**19.65**	14.73	27.97	30.06
+	2-2 / 5-5	CYA – CYA	-	**0.91**	1.65	2.03	1.72
+	2-5 / 5-2	CYA – CYA	-	**0.89**	2.00	2.11	1.76
+	2-2 / 5-5	CYA – CYA*	-	**1.15**	2.36	1.64	1.55
+	2-5 / 5-2	CYA – CYA*	-	**1.94**	4.29	2.07	2.29

*EGF-7/8 fixed with regards to superfold based on EM map

G

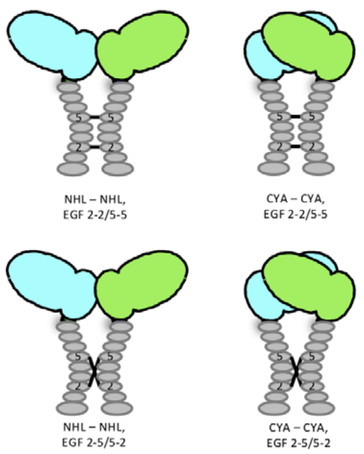

Figure EV5.

 © 2022 The Authors
